# Extracellular vesicles from pristane-treated CD38-deficient mice express an anti-inflammatory neutrophil protein signature, which reflects the mild lupus severity elicited in these mice

**DOI:** 10.3389/fimmu.2022.1013236

**Published:** 2022-10-24

**Authors:** Paula Carrillo-Rodríguez, José-Ángel Robles-Guirado, Adrián Cruz-Palomares, Miguel Ángel Palacios-Pedrero, Elena González-Paredes, Alex Más-Ciurana, Carolina Franco-Herrera, Paloma A. Ruiz-de-Castroviejo-Teba, Antonio Lario, Victoria Longobardo, Laura Montosa-Hidalgo, María M. Pérez-Sánchez-Cañete, María-Mercedes Corzo-Corbera, Sandra Redondo-Sánchez, Ana-Belén Jodar, Francisco J. Blanco, Esther Zumaquero, Ramón Merino, Jaime Sancho, Mercedes Zubiaur

**Affiliations:** ^1^ Department of Cellular Biology and Immunology, Institute of Parasitology and Biomedicine López-Neyra, Consejo Superior de Investigaciones Científicas (IPBLN-CSIC), Granada, Spain; ^2^ Proteomics Unit, Institute of Parasitology and Biomedicine López-Neyra, Consejo Superior de Investigaciones Científicas (IPBLN-CSIC), Granada, Spain; ^3^ Microscopy Facility, Institute of Parasitology and Biomedicine López-Neyra, Consejo Superior de Investigaciones Científicas (IPBLN-CSIC), Granada, Spain; ^4^ Flow Cytometry Unit, IPBLN, CSIC, Granada, Spain; ^5^ Faculty of Physical Sciences, University of Granada (UGR), Granada, Spain; ^6^ Department of Biochemistry, Molecular Biology III, and Immunology, School of Medicine, UGR, Granada, Spain; ^7^ Department of Microbiology, University of Alabama at Birmingham (UAB), Birmingham, AL, United States; ^8^ Department of Cell & Molecular Signalling, Institute of Biomedicine and Biotechnology of Cantabria (IBBTEC), University of Cantabria (UC) and Consejo Superior de Investigaciones Científicas (CSIC), Cantabria, Spain

**Keywords:** extracellular vesicles, inflammation, pro-resolving neutrophil-signature, lupus, CD38, annexin-1, CD47, protein corona

## Abstract

In CD38-deficient (**Cd38^-/-^*)* mice intraperitoneal injection of pristane induces a lupus-like disease, which is milder than that induced in WT mice, showing significant differences in the inflammatory and autoimmune processes triggered by pristane. Extracellular vesicles (EV) are present in all body fluids. Shed by cells, their molecular make-up reflects that of their cell of origin and/or tissue pathological situation. The aim of this study was to analyze the protein composition, protein abundance, and functional clustering of EV released by peritoneal exudate cells (PECs) in the pristane experimental lupus model, to identify predictive or diagnostic biomarkers that might discriminate the autoimmune process in lupus from inflammatory reactions and/or normal physiological processes. In this study, thanks to an extensive proteomic analysis and powerful bioinformatics software, distinct EV subtypes were identified in the peritoneal exudates of pristane-treated mice: 1) small EV enriched in the tetraspanin CD63 and CD9, which are likely of exosomal origin; 2) small EV enriched in CD47 and CD9, which are also enriched in plasma-membrane, membrane-associated proteins, with an ectosomal origin; 3) small EV enriched in keratins, ECM proteins, complement/coagulation proteins, fibrin clot formation proteins, and endopetidase inhibitor proteins. This enrichment may have an inflammation-mediated mesothelial-to-mesenchymal transition origin, representing a protein corona on the surface of peritoneal exudate EV; 4) HDL-enriched lipoprotein particles. Quantitative proteomic analysis allowed us to identify an anti-inflammatory, Annexin A1-enriched pro-resolving, neutrophil protein signature, which was more prominent in EV from pristane-treated *Cd38^-/-^* mice, and quantitative differences in the protein cargo of the ECM-enriched EV from *Cd38^-/-^* vs WT mice. These differences are likely to be related with the distinct inflammatory outcome shown by *Cd38^-/-^* vs WT mice in response to pristane treatment. Our results demonstrate the power of a hypothesis-free and data-driven approach to transform the heterogeneity of the peritoneal exudate EV from pristane-treated mice in valuable information about the relative proportion of different EV in a given sample and to identify potential protein markers specific for the different small EV subtypes, in particular those proteins defining EV involved in the resolution phase of chronic inflammation.

## 1 Introduction

Systemic lupus erythematosus (SLE) is a chronic multi-organ inflammatory disease in which autoantibodies against nucleic acid–protein complexes, such as chromatin and ribonucleoproteins (RNPs), cause disease by forming immune complexes that deposit in target tissues. A type I IFN–dependent lupus syndrome closely resembling human SLE develops in BALB/c, C57BL/6 (B6), and other strains of mice with chronic inflammation following an intraperitoneal injection of pristane (2,6,10,14-tetramethylpentadecane) (1–3). Autoantibody production and glomerulonephritis in pristane-induced lupus requires Toll-like receptor 7 (TLR-7)–mediated type I IFN production driven by the transcription factors IFN regulatory factor 5 (IRF-5) and IRF-7 (3).

Intraperitoneal injection of pristane to CD38 deficient (*Cd38^-/-^*) mice provokes a milder inflammatory reaction than in WT B6 mice (4). We show that pristane-treated *Cd38* ^−/−^ mice, but not *Art2* ^−/−^ mice, had impaired recruitment of peritoneal exudate cells (PECs) compared to WT mice, particularly Ly6C^hi^ monocytes and Ly6C^lo^ monocytes/macrophages and Ly6G^+^ neutrophils, and that this decreased cellularity in the peritoneal cavity (PC) was not due to a defective chemotaxis of these cells but instead due to decreased apoptosis-mediated cell death of PECs, characterized by defective caspase-3 activation and decreased levels of phosphorylated Akt (4). We identified Ly6C^hi^ monocytes and Ly6C^lo^ monocytes/macrophages as the apoptosis-resistant cell populations. Given the implication of CD38 in the activation of the cation channel TRPM2 *via* the production of cADPR/ADPR (5, 6), we analyzed the phenotype of pristane-treated *Trpm2* ^−/−^ mice and observed similar protection from apoptosis-mediated death of PECs in these mice (4). Furthermore, the production of anti-single-stranded DNA and anti-nuclear RNP (nRNP) autoantibodies, the development of glomerulonephritis, and the expression of IFN-I-stimulated genes (ISGs) were also greatly attenuated in pristane-treated *Cd38* ^−/−^ mice. Hence, our data suggest that CD38 deficiency protects mice from pristane-induced lupus in a TRPM2-dependent manner by reducing the number of intraperitoneal apoptotic cells (4), which are the primary source of autoantigens in this murine model of SLE (7).

Generation of extracellular vesicles (EV) is a common property of cells. Intensive research of the last decade has revealed a multitude of different biologic—both physiologic and pathologic—effects of EV (8, 9). Our group provided the first experimental evidence that CD38 is expressed on the surface of secreted exosomes derived from lymphoblastoid B cells (10). Exosomic CD38 is associated with the signaling molecules CD81, Hsc-70 and Lyn (10). More recent work has proved the presence of complete adenosinergic machinery (CD38, CD203a, CD39 and CD73) on EV isolated from myeloma multiple or lymphoblastoma patients (11, 12). Tumor-derived EV are capable of generating of adenosine (ADO) starting from ATP (through the action of CD39 and CD73) or NAD^+^ (through the action of CD38, CD203a and CD73). ADO produced in the tumor microenvironment is able to interact with ADO receptors on T lymphocytes and NK cells, shutting down anti-tumor immune response. Moreover, tumor-derived EV, upon distribution by circulatory stream, may vehicle peripheric effect, modulating immune response by cells expressing ADO receptors (T and B lymphocytes, NK cells and monocytes/macrophages) (13).

Many cell types of the innate immune system have been shown to release EV. EV released from monocytes/macrophages can exert several different functions. First, these EV were shown to cause inflammation-induced programmed cell death in vascular smooth muscle cells *via* transfer of functional pyroptotic caspase-1. Subsequently, it was shown that macrophage-derived EV could induce differentiation of naïve monocyte recipient cells to macrophages. In addition, EV released by macrophages contain MHC class II and co-stimulatory molecules and, similar to DC-derived EV, can play a role in antigen presentation (14). The number of neutrophil-derived EV was reported to become significantly elevated in various pathologic conditions (14), and neutrophils produce proinflammatory or anti-inflammatory extracellular vesicles depending on the environmental conditions (15). PMN-derived EV induced the secretion of the anti-inflammatory cytokine TGFβ from monocytes or DCs and decreased the release of the inflammatory cytokines IL-8, IL-6 and TNFα. They also promoted the phagocytosis of apoptotic PMN and the release of pro-resolving mediators from macrophages. The anti-inflammatory protein Annexin A1 from PMN-EV impaired the adhesion of leukocytes to ECs, while EV produced during the process of PMN extravasation seemed to enhance the endothelial barrier function. PMN-EV may also display a pro-thrombotic function by the encapsulation of platelet-activating factor, combined with the exposure of activated Mac-1 (CD11c/CD18) integrin and tissue factor (14).

Given the massive recruitment of pro-inflammatory Ly6C^hi^ monocytes and neutrophils to the PC of pristane-treated mice, and the distinct outcome in WT versus CD38-deficient mice (4), we have compared the protein composition and protein abundance of the EV present in the peritoneal exudates from pristane-treated WT vs CD38-deficient mice. To this end, 3 distinct sets of EV were isolated from crude EV preparations by size exclusion chromatography (SEC) procedures and proteins were identified and quantified by LC-MS/MS. Our results demonstrate the power of a hypothesis-free and data-driven approach to transform the expected heterogeneity of the peritoneal exudate EV from pristane-treated mice in valuable information about the relative proportion of different EV in a given sample and to identify potential protein markers defining the different types of EV, in particular those proteins defining EV involved in the resolution phase of chronic inflammation.

## 2 Material and methods

### 2.1. Mice and pristane treatment

*Cd38^-/-^* mice were backcrossed for 12 generations to the C57BL/6 J (B6) background (16), bred and maintained under specific pathogen-free conditions at the IPBLN-CSIC Animal Facility in Granada, Spain; as previously described (4). Wild-type C57BL/6 J (B6 WT) (RRID : IMSR_JAX:000664) mice were purchased from Charles River (Barcelona, Spain). *Cd38^-/-^* onto a B6 background (RRID : IMSR_JAX:003727) were provided by Dr. Frances Lund (UAB, Birmingham, USA). All the mice were female, due to the prevalence of SLE in this gender. The mice studied in these experiments were between 7 and 14 weeks of age.

Experimental mice received a single dose of 0.5 ml pristane (2, 6, 10, 14-tetramethylpentadecane (TMPD); sterile filtered and tested for the absence of endotoxins, (P1403,Sigma-Aldrich) (4),. All experimental procedures involving animals at IPBLN-CSIC were approved by the Institutional Animal Care and Use Committee, which follows the ARRIVE guidelines (17), in accordance with the U.K. Animals (Scientific Procedures, Act, 1986) and associated guidelines (EU Directive 2010/63/EU for animal experiments).

### 2.2 Peritoneal exudates cells

PECs were harvested from mice under sterile conditions, after the sacrifice, at 2 weeks post-pristane treatment. Mice were sacrificed by inhalation of CO_2_. The peritoneal exudates (PE) from pristane-treated mice were obtained after i.p. injection of 5 ml of 1x Ca^2+^ and Mg^2+^ free PBS, pH 7.6, 0.5% BSA, 2 mM EDTA, 0.22 μm sterile filtered; followed by a gentle massage of the abdominal cavity. We collected PE containing PECs and EV from the abdominal cavity by means of a small incision, with p1000-pipette sterile tips, into separate 15 ml sterile Falcon type tubes for each mouse. 15 ml-tubes were kept on ice, next they were spun down at 200 x g, 4°C for 5 min, to isolate PECs in the cellular pellet and EV in the supernatant. The EV containing supernatants were transferred to new 15 ml conical tubes. Cellular pellets containing PECs were treated (1:2, v:v) with Ammonium Chloride Solution, 5 min on ice; followed by 8.5 ml of DMEM complete medium, 0.22 μm sterile filtered, and containing 5% heat inactivate Fetal Bovine Serum (FBS), pen:strep, 100 units/ml/10 μg/ml; 10 mM Hepes, 1X L glutamax, 2mM EDTA. PECs were spun down at 360 x g, 4°C for 5 min, the supernatant aspirated and fresh DMEM complete medium was added to resuspend the cellular pellet.

### 2.3 Cellular lysates

Isolated PECs were count and viability was analyzed on a hemocytometer with 1:1 Trypan blue solution (Sigma-Aldrich Cat# T8154). Cells were washed thoroughly three times with cold 1x Ca^2+^ and Mg^2+^ free PBS, pH 7.6, 2 mM EDTA (filter sterilized in 0.22 mm filter); by centrifugation at 360 x g, 4°C for 5 min, the supernatant aspirated and fresh 1 x PBS-2mM EDTA was added to resuspend the cellular pellet. Cells were lysed 30 min on ice, as previously described (10): 100 microliters of 1 x lysis buffer per 10 million cells. The 1x lysis buffer (1xLB) composition is as follows: 150 mM NaCl, 20 mM HEPES (pH: 7.6), 50 mM sodium fluoride, 1 mM EGTA, 1% NP-40, and small peptide inhibitors (SPI) (stock, 50X) (10); 1 mM sodium orthovanadate, 10 mM iodoacetamide, and 1 mM PMSF, as phosphatases inhibitors; plus 0.25 μM trichostatin and 5 mM nicotinamide as acetylases inhibitors. Micro BCA Protein Assay (ThermoFisher Cat# 23235) was used for the analysis of the protein concentration of 17,000 x g-clarified PECs lysates.

### 2.4 EV isolation by size-exclusion chromatography

Supernatants containing EV’s were sequentially centrifuged twice 10 min at 4°C, first at 200 x g, and second at 1,000 x g. After each centrifugation, supernatants containing EV’s were transferred to new 15-ml sterile Falcon type tubes and pellets were discarded. Supernatants containing EV from the identical mice’s strain within the same experiment were combined into a new 50-ml sterile Falcon type tube. The pool of supernatants containing EV was filtered through 0.22 μm Steriflip-GP Sterile Filter Unit (Millipore-Merck). The filtrate was concentrated on a Mr 50,000 MW-cutoff VivaSpin concentrator to a final volume between 1 to 1.5 ml.

**Figure 1:**
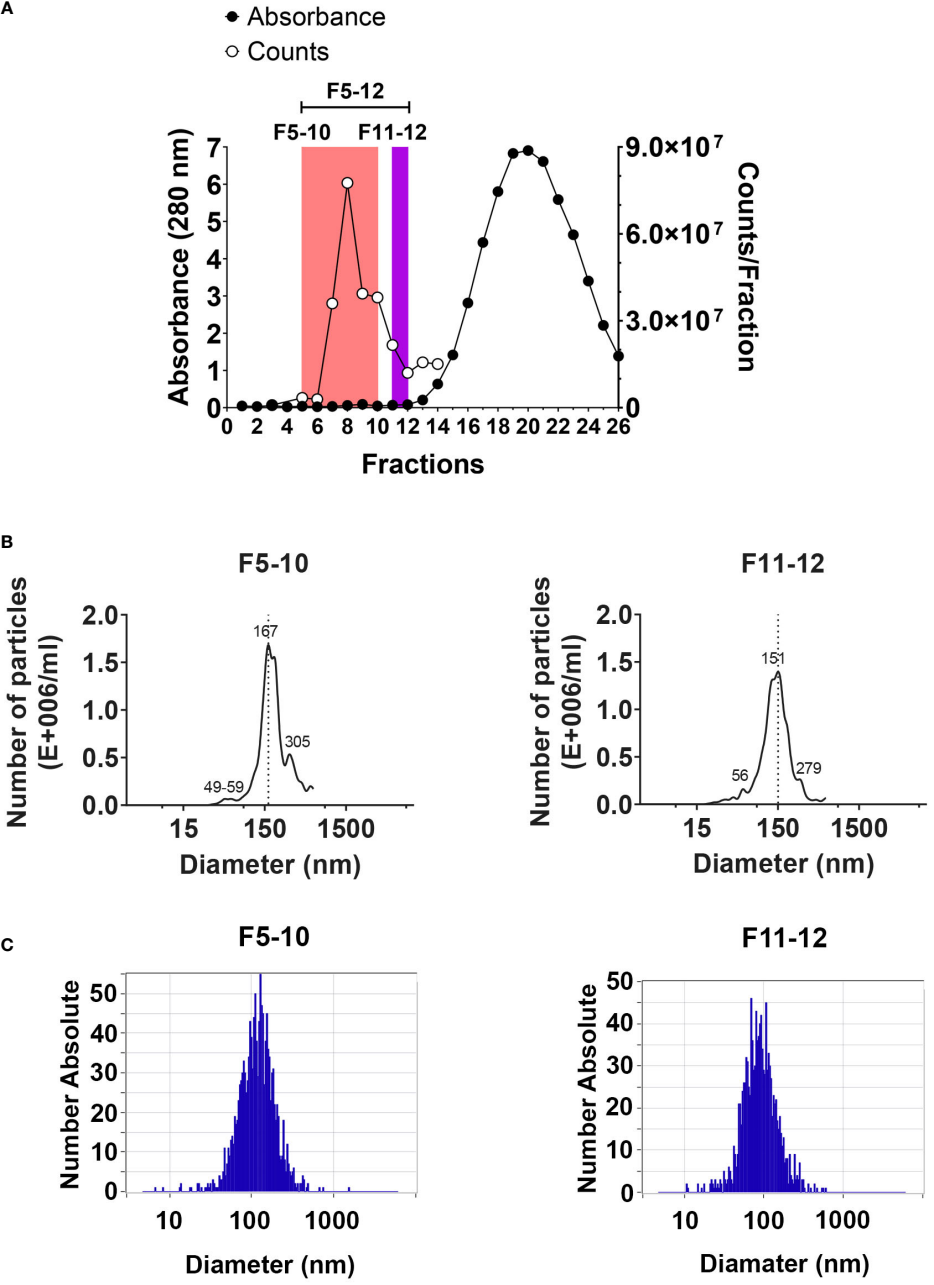
EV isolation by size exclusion chromatography. **(A)** Protein profile detected by absorbance at 280 nm using a Nanodrop spectrophotometer (left Y axis). The number of particles per fraction was assessed by cell flow cytometry using the ZE5 cell analyzer (right Y axis). **(B)** Size distribution of particles in pooled F5-10 and F11-12 fractions from qEV columns using the NanoSight LM10. **(C)** The same as **(B)** using the ZetaView PMX-120.

EV isolation was performed by SEC in qEV sepharose columns (qEVoriginal/70nm, Izon, https://izon.com/) following IZON´s protocol. The qEV columns were equilibrated with Ca^2+^ and Mg^2+^ free PBS, pH 7.6, 5 mM EDTA, and sterile filtered by means of 0.22 µm (PBS-E). Column flow was maintained between 0.8 to 1.2 ml/min. EV sample volume loaded was ranged between 0.5 to 0.9 ml. 0.5 ml fractions were collected into 1.5-ml eppendorf tubes. The protein concentration in each fraction was estimated by mean of the absorbance at 280 nm in a Thermo Scientific Nanodrop 2000 microvolume spectrophotometer (RRID : SCR_018042). Isolated EV were analyzed from eluted fractions F5 to F12 (**Figure 1A**). Aliquots (20 to 30 microliters) from each fraction were separated for particle counting, and microscopy analysis.

### 2.5 Methanol/chloroform protein precipitation from isolated EV fractions

Isolated EV from fractions F5 to F12 were lysed with 0.1% deoxycholic acid, in 1xLB (see above), 10 min, on ice. Proteins from lysed EV from each fraction were precipitated with methanol/chloroform. After precipitation and drying at room temperature, the precipitate was resuspended in 60 mM octyl D-glucoside (ODG) in 1xLB containing 1% NP-40, described above; next the samples were incubated in a Thermomixer at 900 rpm, for 5 min, at room temperature (RT). Laemmli Sample buffer with β-mercaptoethanol was added to each sample to a final concentration of 1x and 5% respectively. Incubation in the Thermomixer was repeated, followed by heat block incubation at 100°C, 5 min, and 17,000 x g microfuge centrifugation, at RT. Within the same experiment, EV fractions F5 to F12 were joint in a pool and saved at -80°C.

### 2.6 Mass spectrometry proteomics and data processing

Proteins from pooled EV fractions in 1x Laemmli reducing sample buffer were loaded on 4-20% Mini-PROTEAN TGX, Stain-free gels (BioRad Cat# 4568095). Likewise, thirty to 40 microgram of protein from PECs cell lysates in 1x Laemmli sample buffer were also analyzed on 4-20% Mini-PROTEAN TGX, Stain-free gels. Electrophoresis was done at 200 V, in 25 mM Trizma base, 192 mM glycine, pH 8.3, and 0.1% SDS buffer, until the whole sample volume was introduced into the gel matrix. Proteins in the gel were visualized by the exposure of the gel during 5 min to UV light in a GelDoc EZ Image (BioRad). In addition, gels were fixed in 10% Metanol/5%Acetic Acid and stained with SYPRO Ruby (BioRad Cat#1703126) according to the protocol of the manufacturers. An EXQuest Spot Cutter (BioRad) with the PDQuest Advanced program was used to cut the protein bands of the gel. Protein extracts were analyzed by liquid chromatography tandem mass spectrometry (LC‐MS/MS) (Amazon Speed, Bruker) at IPBLN-CSIC Proteomic Facility as described previously (18). Protein identification was done with ProteinScape 4.0 (Bruker) and MASCOT 2.4 (RRID : SCR_014322) data searching using the SwissProt database.

For label-free proteomic quantification, we used the exponentially modified protein abundance index (emPAI) implemented into the MASCOT data searching platform without any additional experimental steps. In order to compare between different samples, it is required to normalize emPAI values from MASCOT search to the sum of all emPAI values. Thus, the protein content in molar fraction percentage (M%) can be calculated using the following formula: Protein content (M%) = emPAI/(∑(emPAI), where ∑(emPAI) is the summation of emPAI values for all the identified proteins (19). Several biological samples per mouse type and three technical replicates per biological sample were analyzed. The fold-change (FC) in protein abundance was calculated by dividing the M% value of and individual protein in *Cd38^-/-^* EV with the cognate value in WT EV. This ratio has been useful to identify which proteins are more or less abundant in *Cd38^-/-^* EV vs WT EV; proteins with a *Cd38^-/-^*/WT ratio > 1 will be more abundant in *Cd38^-/-^* EV, and those with a *Cd38^-/-^*/WT ratio < 1 will be more abundant in WT EV. The log_2_ value of the *Cd38^-/-^*/WT ratio was calculated and finally the absolute fold change calculated as 2 ± 1*log_2_value of the *Cd38^-/-^*/WT ratio (20). We selected a cutoff for biological significance of 1.3 fold in keeping with the fact that the emPAI methodology can reliably classify differences above 1.3-fold differences (21). The mass spectrometry proteomics data have been deposited to the ProteomeXchange Consortium *via* the PRIDE (22, 23) partner repository with the dataset identifier PXD03527 and 10.6019/PXD03527.

We used ClueGO_v2.5.8 (RRID : SCR_005748) (24) and CluePedia_v1.5.8 (RRID : SCR_015784) (25) within the Cytoscape_v3.9.1 software environment (RRID : SCR_003032) (26) for functional enrichment analysis of the lists of identified proteins. Results are visualized as networks in which Gene Ontology (GO) terms and pathways are grouped based on their biological role. CluePedia allows to expand ClueGO terms into nested networks with associated genes. Protein-protein interaction networks were searched using Cytoscape StringApp_v1.7.1 (RRID : SCR_005223) (27). Data analysis was also performed using the R statistical computing environment (RRID : SCR_001905). The shared proteins between EV’s and PECs for each mouse model was calculated by the VennDiagram package (RRID : SCR_002414) and those proteins was transformed to Gene name and analyzed with the EnrichR software (RRID : SCR_001575) (28–30) in R environment. The –log_10_(FDR) was calculated for each database (KEGG, Biological Process, Molecular Function and Cellular Component) and the top 4 terms was selected to display in the graph.

### 2.7 Nanoparticle tracking analysis

The hydrodynamic size distribution of the EV, this is, the particle concentration as a function of the diameter was obtained by Nanoparticle Tracking Analysis (NTA). A NanoSight LM10-HS(GB) FT14 (NanoSight, Amesbury, United Kingdom) equipped with a sample chamber with a 405 nm laser and a high-sensitivity EMCCD camera was used. Video images of the Brownian motion of the particles were captured and analyzed by the NTA 2.3 image analysis software. All samples were measured at least in triplicate at 25°C, with manual shutter, gain, brightness, and threshold adjustments. In selected samples NTA was performed using the ZetaView Nanoparticle Tracking Analyzer (Particle Metrix, RRID : SCR_016647). Samples were measured for size and concentration in scatter mode (488 nm laser) and standard instrument settings following the manufacturer’s instructions.

In addition, the number of particles was also assessed in selected samples using the Bio-Rad ZE5 Cell Analyzer Flow Cytometer (RRID : SCR_019713) that has small particle detection capability (forward scatter (FSC) from the 405 nm laser).

### 2.8 Transmission electron microscopy

Isolated EV samples were analyzed by TEM. The Libra 120 transmission electron microscope (Carl Zeiss, RRID : SCR_016703) was used at the Core Services of the Granada University (https://cic.ugr.es). A uranyl acetate staining was performed, prior to TEM examination.

### 2.9 Immuno-capture assay for isolation/detection of EV

CD63^+^ and CD47^+^ EV were detected by flow cytometry in purified peritoneal EV samples from 2 weeks pristane-treated *Cd38^-/-^* and WT mice by using the *ExoStep*™ mouse kit (Immunostep, reference: MOExoS-25-C9) following the manufacturer’s recommendations (31). Briefly, EV samples were incubated overnight at RT with capture beads (polystyrene micro-particles coated with antibodies against mouse CD63 (clone NVG2), or against mouse CD47 (clone MIAP301), and with discrete fluorescence intensity). After overnight incubation, the bead-bound EV were washed with filtered PBS containing 1% BSA, and recovered using a Magnetic Rack (MagneSphere^®^ Mag. Sep. Stand 12-hole, 12 × 75 mm (Promega, Ref Z5343). After 5 min incubation, supernatant was removed by hand-decanting, then tubes were removed from the magnetic rack and beads were incubated with biotinylated anti-CD9 (Clone MZ3) antibody for 60 min in the dark at 2-8°C, without stirring. Beads were then washed and recovered with the Magnetic Rack as above and incubated with Streptavidin-PE for 30 min in the dark at 2-8°C. Then, beads were washed and recovered with the magnetic rack as above and resuspended in 350 μL of PBS. Then, 2,000 microbeads were immediately acquired using a FACSymphony flow cytometer (BD). Bead-EV population was gated according to their own fluorescence on BB700 vs. APC channels and the quantity of EV was determined by the MFI (median fluorescence intensity) in the PE channel. Relative Fluorescence Intensity (RFI) was calculated as MFI positive/MFI background.

### 2.10 Statistical analysis and reproducibility

Statistical analysis was performed using GraphPad Prism 9 software (GraphPad Prism, RRID: SCR_002798), using statistical tests as indicated in the text. Statistical significance was visualized as: ns = not significant (*P* > 0.05); * = *P* < 0.05; ** = *P* < 0.01; *** = *P* < 0.001; **** = *P* < 0.0001. All experiments have been done using three or more mice and representative images have been chosen for figures.

## 3 Results

### 3.1 Isolation of EV by size exclusion chromatography size distribution

EV were isolated by SEC from peritoneal exudates from mice 2 weeks after the i.p. injection of pristane, as described in Material and Methods using the qEV/70 nm Original (Izon) columns, which are optimized to the higher recovery of EV larger than 110 nm (70-1000 nm optimum recovery range) and less lipoprotein overlap. The protein concentration in each fraction was estimated by mean of the absorbance at 280 nm in a Nanodrop (**Figure 1A**), and the number of particles per fraction was assessed by flow cytometry using the ZE5 cell analyzer, which has the capability of detection of small particle detection capability (**Figure 1A**). Three pooled fractions were further analyzed: F5-12; F5-10 and F11-12. Particle size distribution in F5-10 and F11-12 was evaluated by NTA using the Nanosight LM10 of Malvern (**Figure 1B**), or the Zetaview PMX-120 of Particle Metrix (**Figure 1C**). Using the Nanosight LM10, F5-10 particles showed a maximum peak at 167 nm, and F11-12 at 151 nm. Moreover, both pooled samples showed 2 minor peaks. One at about 55 nm and the second one at 280-305 nm range. By using the Zetaview, the median size of F5-10 particles was 115 nm, where 74% showed a diameter of 131 nm and 23% had an 81 nm diameter. In contrast, F11-12 particles showed a median value of 86 nm, where 93% showed a diameter of 88 nm. Therefore, the size of the particles fits well with the size of the so-called small EV (less than 200 nm).

### 3.2 Transmission electron microscopy analysis of pooled SEC fractions

**Figure 2:**
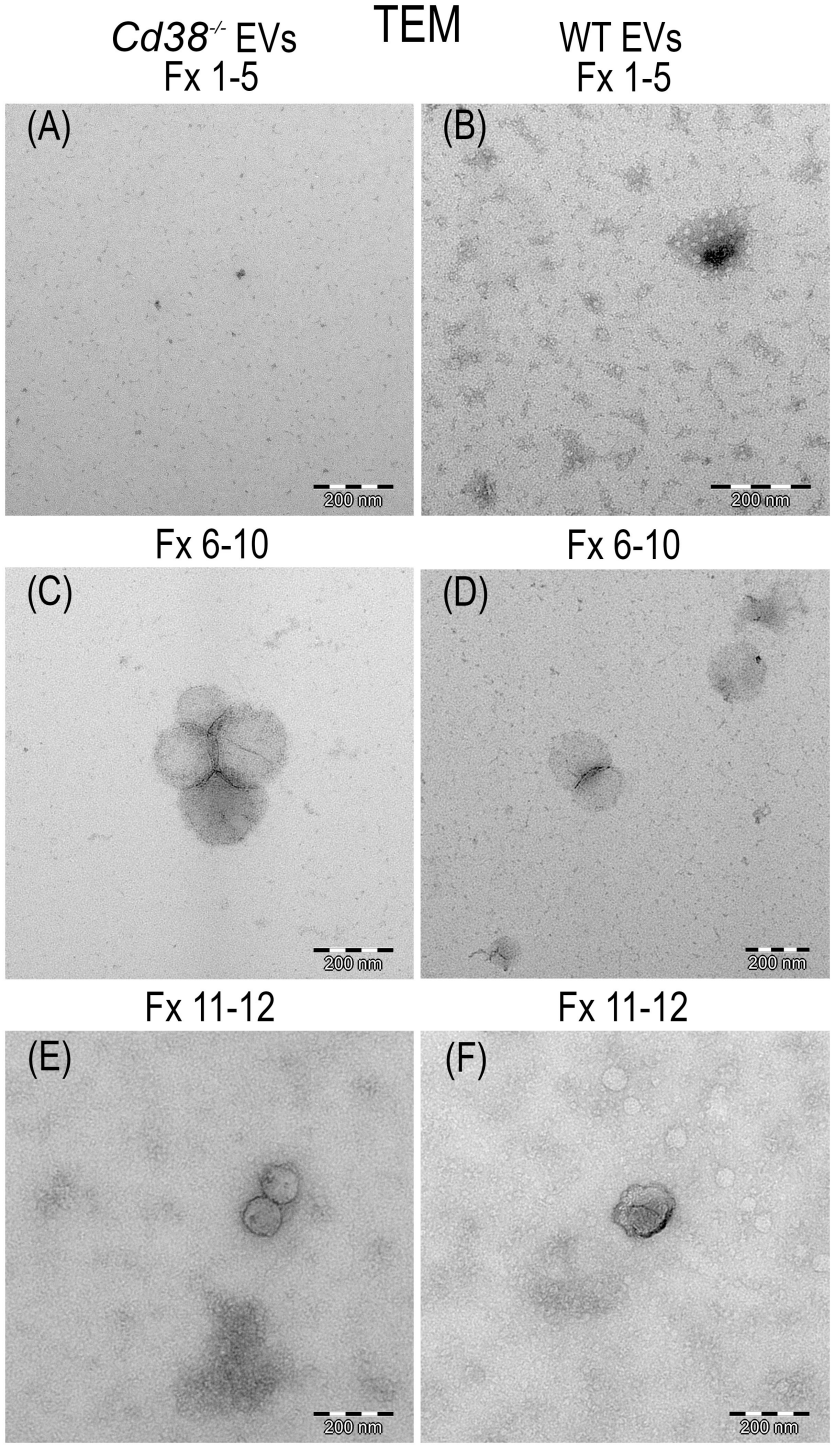
Transmission electron micrograph photos of SEC pooled fractions of supernatants from peritoneal exudates of 2wks pristane-treated *Cd38^-/-^* mice **(A, C, E)** or WT mice **(B, D, F)**. F1-5 showed only grid background **(A, B)**. F6-10, vesicles were easily identified **(C, D)**. F11-12, vesicles of smaller size than in F6-10 were more frequently observed. EV, negatively stained, appeared dark; LPPs appeared white.

TEM has nanometer resolution and it has been used to distinguish single EV from non-EV particles. **Figure 2** shows the result of the TEM analysis of the pooled fractions F1 to F5; F6 to F10 and F11 to F12 from peritoneal EV isolated by SEC as described above. Vesicles were identified in both F6-10 and F11-12, but not in F1-5. The size of the vesicles seemed to be larger in F6-10 versus F11-12, in agreement with the NTA data (**Figure 1C**). Note that the cup-shaped vesicles (**Figure 2F**) are a commonly reported TEM artifact of isolated exosomes (32). To note is the presence in F11-12 (**Figure 2F**) of small round vesicles less than 30 nm without visible membranes that may correspond to lipoprotein particles (LPPs), which most of them are smaller than EV, and appear white in TEM preparations (33).

### 3.3 Distinct proteomic profiling and associated functional terms in EV from CD38-deficient mice

**Figure 3:**
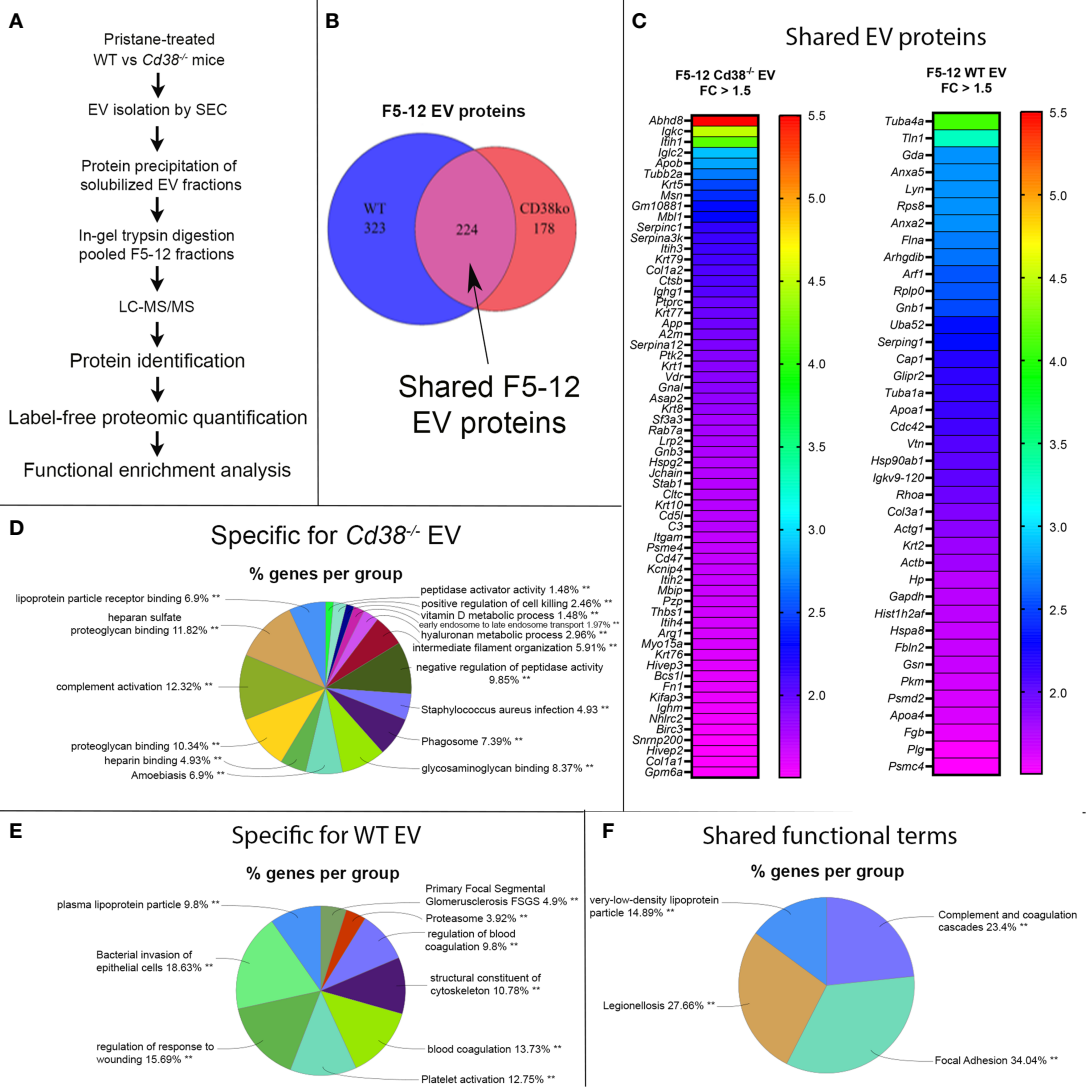
**(A)** General workflow for LC-MS-based EV protein identification, protein quantification and functional enrichment of samples from pristane-treated WT vs *Cd38^-/-^* mice. **(B)** Venn diagram of proteins identified in peritoneal F5-12 *Cd38^-/-^* EV versus F5-12 WT EV from pristane-treated mice. In red, 178 proteins unique for *Cd38^-/-^* EV. In blue, 323 proteins unique for WT EV. In purple, shared proteins identified in both EV types. **(C)** Heat-maps showing increased protein abundance in F5-12 *Cd38^-/-^* EV relative to F5-12 WT EV (left), and increased protein abundance in F5-12 WT EV relative to F5-12 *Cd38^-/-^* EV (right). Absolute Fold-Change (FC) (Y-axis) in **(C)** was calculated as described in Material and Methods. A cutoff of 1.3 fold was selected for biological significance. For clarity only proteins with an absolute FC ≥ 1.5 are represented. For clarity the gene names of the proteins are shown. **(D)** Pie chart showing the main functional groups specific to F5-12 *Cd38^-/-^* EV. **(E)** Main functional groups specific to F5-12 WT EV. **(F)** Pie chart representing the functional groups with equal proportions of proteins from F5-12 *Cd38^-/-^* EV and F5-12 WT EV, therefore shared by both EV types. The level of significance of the terms is shown as ** (*P* value < 0.001).

In this study we performed the comparative analysis of the proteins identified by MS/MS in isolated EV from peritoneal exudates of pristane-treated *Cd38^-/-^* and WT mice (see workflow in **Figure 3A**). The compilation list of identified proteins in the F5-12 subset includes 402 proteins in *Cd38^-/-^* EV and 547 proteins in WT EV. Each protein has an associated mean value of molar percentage calculated with the individual molar percentages obtained in each MS/MS run or replicate; 13 replicates were conducted with WT mice and 9 replicates with *Cd38^-/-^* mice. Since a given protein was not always identified in each run, each protein has a value of “n” or sample size less than or equal to 13 in the case of WT EV and less than or equal to 9 in the case of *Cd38^-/-^* EV. The protein analyses have been approached from three perspectives: (i) proteins that are expressed only in *Cd38^-/-^* EV, (ii) proteins that are expressed only in WT EV, and (iii) proteins that are expressed in both groups (common proteins), establishing levels of increased abundance and decreased abundance in *Cd38^-/-^* EV relative to WT EV. **Figure 3B** corresponds to a Venn diagram, made using the eulerr program (34), which represents the number of proteins in each group: 178 proteins unique to *Cd38^-/-^* EV in red, 323 proteins unique to WT EV in blue, and 224 proteins common to both groups in purple. Each of these proteins has an associated identifier, UniProtKB AC/ID, unique to the protein and the species, and these identifiers (ID) can be analyzed by using different applications.

Peritoneal exudate F5-12 WT EV and F5-12 *Cd38^-/-^* EV from 2-wk pristane-treated mice shared 224 of the identified proteins by MS/MS (**Figure 3B**). This allowed the comparison of the relative abundance of these proteins between the 2 experimental groups. The comparison of abundance levels was performed by virtue of the molar percentage fraction (M%) of the identified proteins in WT EV and *Cd38^-/-^* EV (see Material and Methods). The fold-change (FC) in protein abundance was calculated by dividing the M% value of and individual protein in *Cd38^-/-^* EV with the cognate value in WT EV. This ratio has been useful to identify which proteins are more or less abundant in *Cd38^-/-^* EV vs WT EV; proteins with a *Cd38^-/-^*/WT ratio > 1 will be more abundant in *Cd38^-/-^* EV, and those with a *Cd38^-/-^*/WT ratio < 1 will be more abundant in WT EV. The log_2_ value of the *Cd38^-/-^*/WT ratio was calculated and finally the absolute fold change calculated as 2 ± 1*log_2_value of the *Cd38^-/-^*/WT ratio (20). We selected a cutoff for biological significance of 1.3 fold in keeping with the fact that the emPAI methodology can reliably classify differences above 1.3-fold differences (21).

Taking into account the above criteria, 88 proteins showed increased abundance in F5-12 *Cd38^-/-^* EV (cluster #1, **Figure 3C**, left), and 52 proteins had increased abundance in F5-12 WT EV (cluster #2, **Figure 3C**, right). Functional enrichment analysis of GO terms and pathways of proteins Cluster #1 versus Cluster #2 was performed using ClueGO + CluePedia applications (**Supplemental Figure 1**). After GO Term Fusion, and P value significance selection criteria, 81 out of 140 proteins were functionally annotated and associated to 65 representative Terms and Pathways. Terms were functionally grouped based on shared genes (kappa score) and the most significant term defined the name of the group. The functional groups associated with each cluster are represented in pie charts (**Figures 3D, E**), where the percentage of found proteins per group are shown. **Figure 3F** represents those functional groups with equal proportions of proteins from the 2 clusters.

### 3.4 Identification of CD47 and integrins CD11b and CD18 among the proteins showing increased abundance in peritoneal EVs

**Figure 4:**
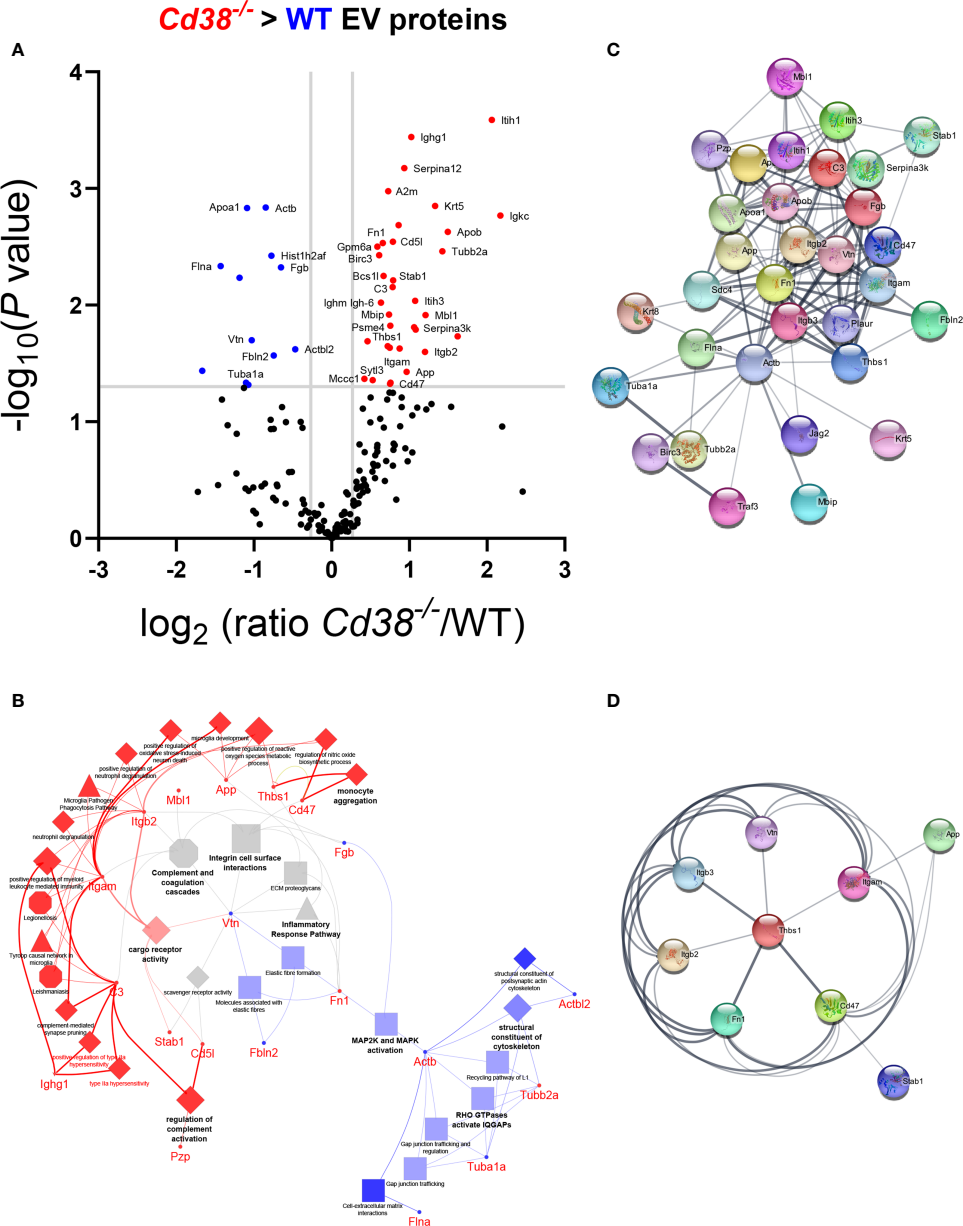
**(A)** Volcano plot of the common proteins to F5-12 *Cd38^-/-^* EV and F5-12 WT EV. The grey horizontal line separates the statistically significant proteins with a pV < 0.05 from the non-significant proteins. Vertical grey lines are indicative of the absolute 1.3 fold-change limit. Red dots indicate proteins in *Cd38^-/-^* EV with an absolute abundance greater than 1.3 and pV < 0.05. Blue dots indicate proteins in WT EV with an absolute abundance greater than 1.3 and pV < 0.05. Black dots are the proteins with no statistically significant differences. The gene names of the proteins with significant increased abundance is shown. **(B)** Network of GO terms and signaling pathways generated from the list of 46 common proteins to F5-12 *Cd38^-/-^* and F5-12 WT EV that showed significant increased abundance in the Volcano plot of panel **(A)**. Two protein lists (clusters) were compared: Proteins with increased abundance in *Cd38^-/-^* EV (cluster #1) versus proteins with increased abundance in WT EV (cluster #2). The network has been created using the ClueGO and Cluepedia applications of Cytoscape 3.9.0, with the ontologies GO Biological Process, Immune System Process, Molecular Function, KEGG, REACTOME Pathways and Wiki Pathways; pV < 0.05. From the 2 proteins clusters that were compared, 3 categories of functional terms were generated: terms and genes specific for F5-12 *Cd38^-/-^* EV (red), terms and genes specific for F5-12 WT EV (blue), and common terms (grey). The node size is indicative of the term statistical significance. Terms or pathways are considered specific for a given cluster when >60% of the proteins are originating from one of the clusters. The terms with the highest statistical significance within each functional group are highlighted in bold and defines the name of the functional group. Gene names instead of protein names or Uniprot IDs are shown in ClueGO and String apps by default. **(C)** Network of protein-protein interactions created with 35 out of the 46 proteins showing significant increased abundance in either F5-12 *Cd38^-/-^* EV or in F5-12 WT EV. This network has been created with the StringApp within Cytoscape with an interaction confidence score of 0.4. **(D)** First protein neighbors interacting with CD47. All these proteins showed increased abundance in *Cd38^-/-^* EV relative to WT EV.

To deepen the statistical processing of common protein data and obtain reliable candidate biomarkers, proteins were analyzed using the Multiple t-test within the GraphPad Prism software with a threshold for P value comparisions of alpha = 0.05. With these data, a Volcano Plot chart (**Figure 4A**) was constructed and the 46 proteins which showed statistically significant differences were selected for further studies. Then, the proteins were analyzed with ClueGO + CluePedia applications in Cytoscape to understand their functions. 2 clusters were compared, cluster #1, proteins with increased abundance in *Cd38^-/-^* EV and cluster #2, proteins with increased abundance in WT EV, using the following ontologies: Biological Process, Molecular Function, Immune System Process, KEGG, REACTOME Pathways and Wiki Pathways (**Figure 4B**). Some terms that were specifically associated with *Cd38^-/-^* EV play important roles in the development of SLE such as Monocyte Aggregation, Regulation of Complement Activation and Positive Regulation of Myeloid Leukocyte Mediated Immunity. Likewise, terms such as MAP2K and MAPK activation or Structural Constituent of Cytoskeleton were preferentially associated with WT EV, while Inflammatory Response Pathway, Complement and Coagulation Cascades, and Integrin Cells Surface Interactions were equally represented in both *Cd38^-/-^* and WT EV.

The common proteins to F5-12 *Cd38^-/-^* EV and F5-12 WT EV, were also analyzed for protein-protein interactions using the StringApp application within Cytoscape. 35 out of the 46 common proteins showed positive interactions (**Figure 4C**). A closer view highlights the importance of CD47 and *Thbs1* in nucleating some of these interactions (**Figure 4D**). CD47 is a five-transmembrane domain protein (35), which delivers a “don’t eat me signal” upon binding to the Signal-regulatory protein alpha (SIRPα) receptor on myeloid cells (36). Another ligand for CD47 is Thrombospondin-1 (TSP-1) encoded by *Thbs1*. TSP-1 is strongly expressed in neutrophils and is secreted in response to inflammation, promoting the resolution of the inflammatory process and facilitating phagocytosis of damaged cells (37). In this network of interactions participated the integrins *Itgam* and *Itgb2*, which correspond to the proteins CD11b and CD18, respectively. CD11b and CD18 as a heterodimer (Mac-1) is implicated in various adhesive interactions of monocytes, macrophages and granulocytes and plays an important homeostatic role in inflammation by accelerating the elimination of extravasated neutrophils (38).

### 3.5 Increased abundance of neutrophil-derived proteins and associated functional terms

**Figure 5:**
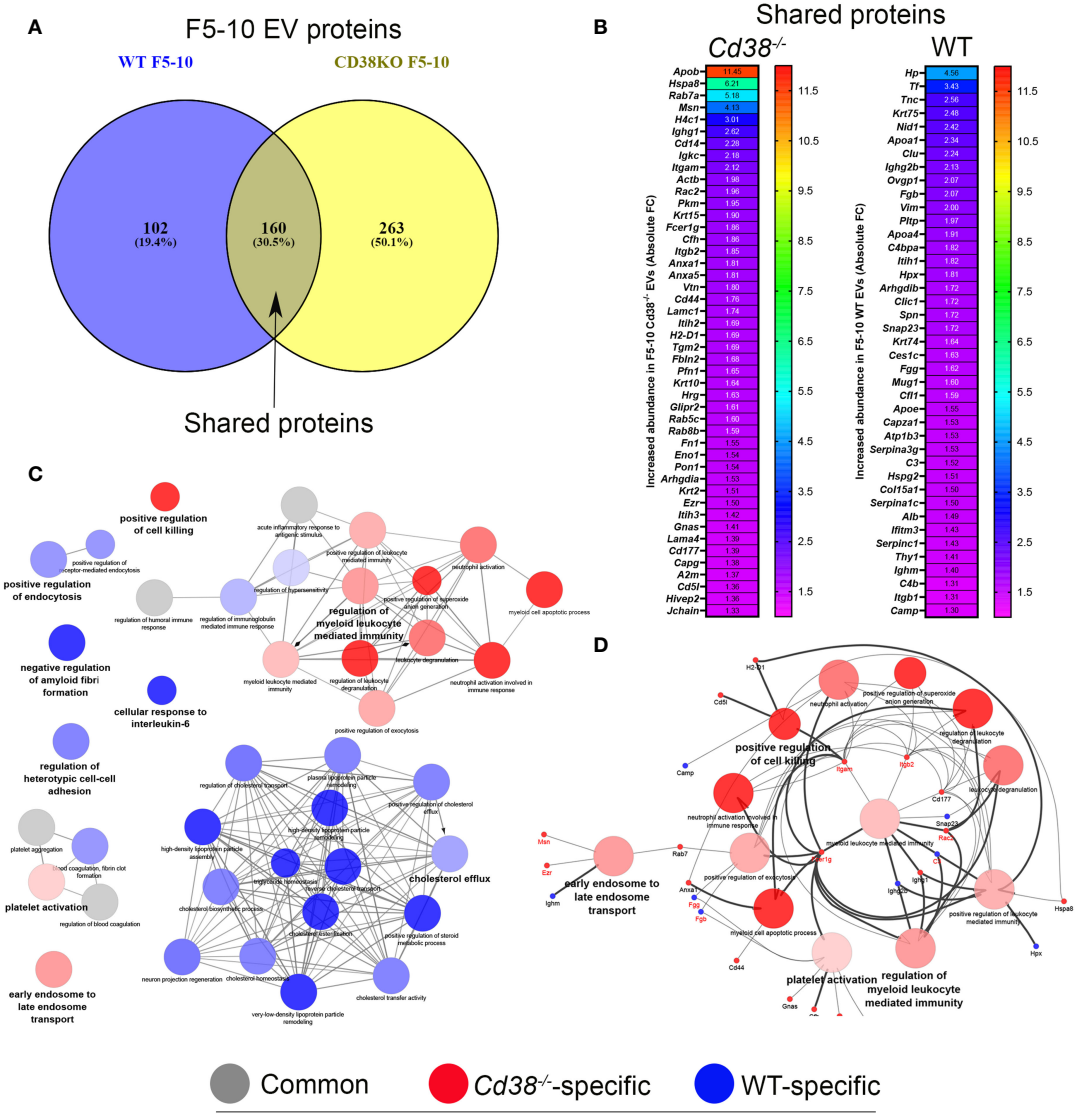
**(A)** Venn diagram highlighting the number of shared proteins identified in both peritoneal F5-10 *Cd38^-/-^* EV and F5-10 WT EV from pristane-treated mice. **(B)** Heat-maps showing the increased protein abundance in F5-10 *Cd38^-/-^* EV relative to F5-10 WT EV (left), or the increased protein abundance in F5-10 WT EV relative to F5-10 *Cd38^-/-^* EV (right). The absolute Fold-Change (FC) (X-axis) in **(B)** was calculated as described in Material and Methods. Only proteins with a cutoff ≥1.3 are shown. For clarity the gene names of the proteins are shown. **(C)** Network of terms generated with the proteins common to F5-10 *Cd38^-/-^* EV and F5-10 WT EV, which showed an absolute FC *≥* 1.3. The network has been created using the ClueGO and Cluepedia applications of Cytoscape, with the GO Biological Process; pV < 0.05. From the 2 proteins clusters that were compared, 3 categories of terms were generated: terms specific for F5-10 *Cd38^-/-^* EV (red), terms specific for F5-10 WT EV (blue), and common terms (grey). The node size is indicative of the term statistical significance. Terms are considered specific for a given cluster when >60% of the proteins are originating from one of the clusters. The term with the highest statistical significance within each functional group is highlighted in bold and defines the name of the functional group. **(D)** Network highlighting the genes associated with the 13 functional terms specific for F5-10 *Cd38^-/-^* EV.

The TEM and NTA analyses indicated that the F11-12 pool contained smaller EV than F5-10 and lower total number of particles, but the presence of LPPs was more patent than in the F5-10 pool. Therefore, it was of interest to analyze the proteomic profiles of F5-10 and F11-12 separately to get more insight about the protein composition and functional capabilities of these apparently distinct EV, according with their sizes. Common proteins to F5-10 *Cd38^-/-^* and F5-10 WT EV were quantitatively analyzed following a similar strategy to section 3.3. Of the 160 shared proteins (**Figure 5A**), 49 showed increased abundance above 1.3-fold in F5-10 *Cd38^-/-^* EV (**Figure 5B**), and 45 had increased abundance in F5-10 WT EV (**Figure 5B**). These 2 sets of proteins were analyzed with ClueGO + CluePedia apps using GO Biological Process. The data showed that 13 GO terms were specific to F5-10 *Cd38^-/-^* EV, 23 terms to F5-10 WT EV, and 4 terms were shared by the 2 clusters (**Figure 5C**). In F5-10 *Cd38^-/-^* EV the main functional group was *Regulation of myeloid leukocyte mediated immunity*, which showed the highest statistical significance. This term grouped 6 additional GO terms, many of them related with neutrophil functions (**Figure 5D**). Indeed, the proteins associated with these terms/pathways are highly expressed in neutrophils. Therefore, the increased abundance of neutrophil-derived proteins and associated functional terms are particularly increased in the F5-10 *Cd38^-/-^* EV pool.

### 3.6 Presence of exosomal and ectosomal markers in peritoneal exudate EV

The larger number of proteins identified in F5-10 *Cd38^-/-^* EV than in F5-10 WT EV suggested differences in functional capabilities, which were not fully addressed upon the analysis of the shared proteins. About 50% of the identified proteins in F5-10 EV were unique to *Cd38^-/-^* EV, while only 19.4% of the proteins were unique to WT EV (**Figure 5A**).

#### 3.6.1 Identification of a set of integrins unique to F5-10 *Cd38^-/-^* EV

**Figure 6:**
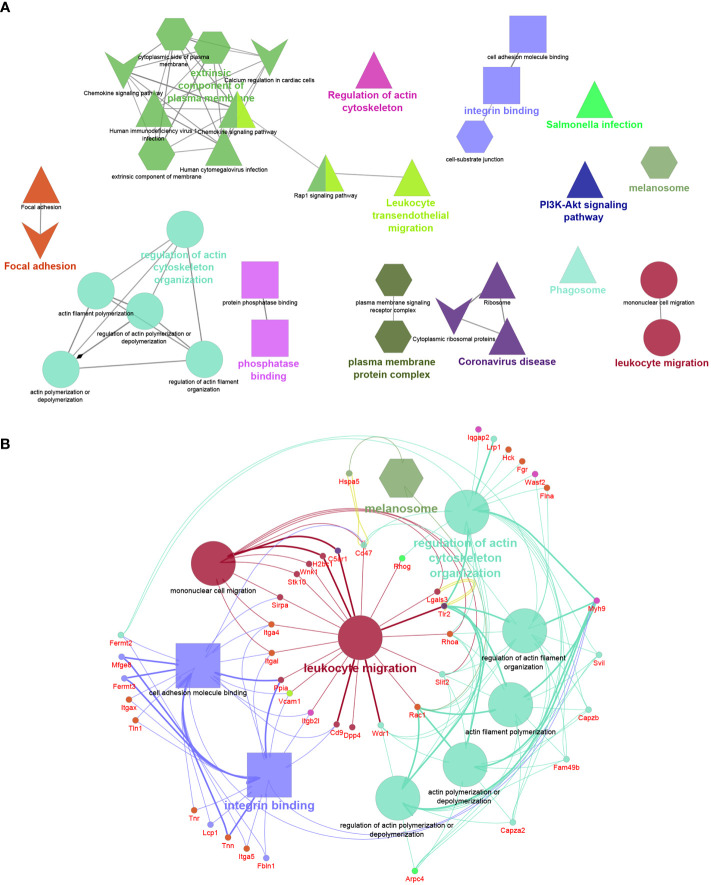
**(A)** Network created with the proteins unique to F5-10 *Cd38^-/-^* EV. **(B)** Network showing the major functional terms and associated genes unique to F5-10 *Cd38^-/-^* EV. The network has been created using the ClueGO and Cluepedia applications of Cytoscape, with the GO Biological Process (circles), GO Cellular Component (hexagons), GO Molecular Function (rectangles), KEGG (triangles), and Wiki Pathways (V shape); pV < 0.05. Terms are functionally grouped based on shared proteins (kappa score) and each functional group is shown with a different color. The term with the highest statistical significance within each functional group is highlighted in bold. The size of the nodes indicates the degree of significance. The most significant term defines the name of the group.

The functional analysis of the 263 proteins unique to F5-10 *Cd38^-/-^* EV yielded a distinct number of functional terms (**Figure 6A**). Among the proteins unique to F5-10 *Cd38^-/-^* EV there were a number of integrins such as *Itgal*, *Itga4*, *Itga5*, and *Itgax*, which envisioned a new landscape of putative EV interactors. In fact, the individual term *Integrin binding* showed the best *P* value (2.66E-10) out of the 37 terms. Per example, *Itgal* endodes CD11a, which in combination with *Itgb2* (CD18) form LFA-1, which is a receptor for ICAM1, ICAM2, ICAM3 and ICAM4 (39), with distinct functions to the pair CD11b/CD18 identified among the shared proteins (**Figure 6B**). *Itgax* encodes the protein CD11c, which in association with CD18 binds to fibrinogen. It is expressed in DCs and macrophages (40). VCAM1, which was also identified within this group, interacts with ITGA4/ITGB1 on leukocytes, and mediates both adhesion and signal transduction. Indeed, these integrins participate in many of the major functional groups identified (**Figure 6B**). It is worth noting that the protein abundance of these integrins is far below the abundance levels of CD11b and CD18 (data not shown), suggesting release in distinct EV (41).

#### 3.6.2 Identification of the exosome markers CD9 and galectin-3 and the EV marker CD47 in F5-10 *Cd38^-/-^* EV

In contrast, another set of proteins showed similar protein abundances to CD11b and CD18, and are associated with similar functional terms, in particular with *Leukocyte migration*. Thus, the tetraspanin CD9, which is frequently utilized as a bonafide exosome marker, was also identified in F5-10 *Cd38^-/-^* EV. CD9 participates in 2 major functional groups: *Integrin binding* and *Leukocyte migration*, together with CD47 and its ligand *Sirpα* (Signal-regulatory protein alpha-1, or CD172a) (**Figure 6B**). SIRP-α1 mediates negative regulation of phagocytosis, mast cell activation and dendritic cell activation (42–44). *C5ar1* encodes CD88, which is 7-transmembrane G-protein coupled receptor, and participates in 2 functional groups: *Leukocyte migration* and in *Coronavirus disease* (**Figure 6B**). In addition to stimulating chemotaxis, granule enzyme release and superoxide anion production, CD88 stimulates upregulation of expression and activity of the adhesion molecule MAC-1, and of CR1 on neutrophils (45). Uncontrolled activation of the C5a–C5aR1 axis has been associated with a myriad of acute and chronic inflammatory diseases (46), including the inflammatory side of COVID-19 infection (47). Complement C5a induces C5aR1-mediated microvesicular shedding from neutrophils resulting in decreased C5aR1 surface expression on granulocytes and impaired cellular chemotactic and pro-inflammatory neutrophil functions (48). *Lgals3* encodes for Galectin-3 (Mac-2), a non-glycosylated lectin which has been shown in exosomes and EV derived from a number of different cells (49). Galectin-3 directly interacts with the endosomal sorting complex required for transport (ESCRT)–component Tsg101. This interaction facilitates the recruitment of Galectin-3 into newly formed intraluminal vesicles (ILVs) in the multivesicular body (MVB). ILVs are sorted to lysosomes for degradation or released as exosomes into the outer milieu (50). Galectin-3 is also involved in acute inflammatory responses including neutrophil activation and adhesion, chemoattraction of monocytes macrophages, opsonization of apoptotic neutrophils, and activation of mast cells. In this search Galectin-3 participates in *Leukocyte migration* and *Phosphatase binding*. Galectin-3 regulates inflammatory reaction in the mesentery during lupus-like responses induced by pristane (51). *Tlr2* encodes TLR2 that serves as a receptor for peptidoglycan and lipoproteins of Gram-positive bacteria (52), and participates in 8 out of the 14 major functional terms. TLR2 is expressed on regulatory T cells (Tregs), where upon its stimulation enhances survival and proliferation of these cells (53). Regarding autoimmunity, TLR2-deficient B6^lpr/lpr^ mice develop a significantly less severe lupus-like disease than B6^lpr/lpr^, as demonstrated by the reduced intensity of glomerulonephritis and the decrease of autoantibody rates (54). Moreover, TLR2 is required for autoantibody production and development of renal disease in pristane-induced lupus (55). According to the Reactome pathways, TLR2, CD88 and Galectin-3 are involved in neutrophil degranulation.

#### 3.6.3 CD82 and EGFR, markers of classical tetraspanin-positive exosomes, were identified exclusively in F5-10 WT EV

**Figure 7:**
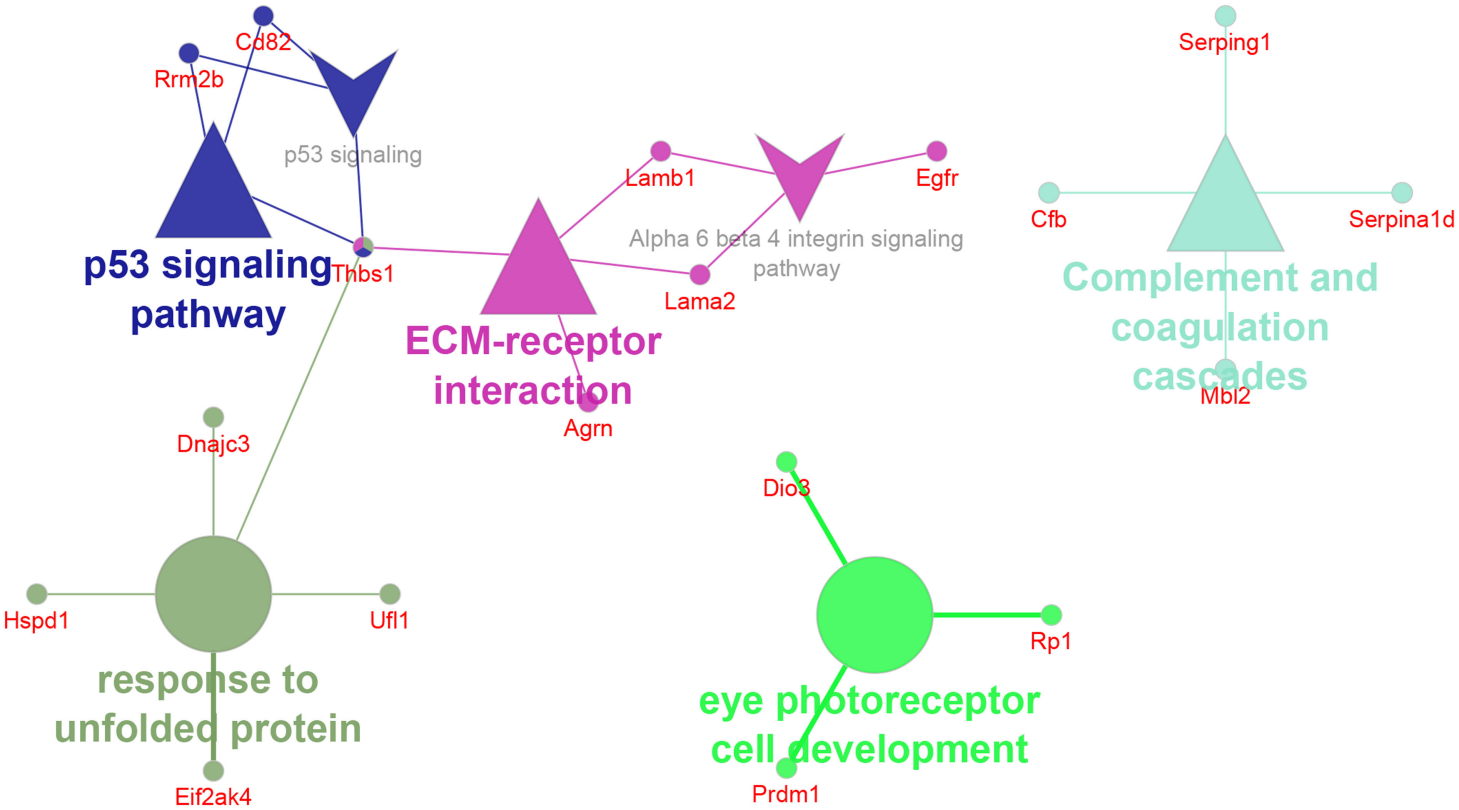
Network of GO terms, pathways and associated genes unique to F5-10 WT EV. The network has been created using the ClueGO and Cluepedia applications of Cytoscape, with the GO Biological Process GO Cellular Component, GO Molecular Function, KEGG and Wiki Pathways; pV < 0.05.

Only a relatively small fraction of the 102 proteins unique to F5-10 WT EV seemed to be integrated in functional terms and networks (**Figure 7**). To note is the identification of the tetraspanin CD82, and the Epidermal Growth Factor Receptor (*Egfr*). Egfr is present in classical tetraspanin-positive exosomes (56, 57). TSP-1 encoded by *Thbs1* was associated with 3 distinct pathways: *p53 signaling pathway*, *ECM-receptor interaction* and *response to unfolded protein*. Another protein associated with the term *ECM-receptor interaction* was Agrin encoded by *Agrn*. This protein has been identified by proteomics in B-cell exosomes (58), and in exosomes from other sources (59, 60). In the context of ECM-receptor interaction, Agrin interacts with integrin β1 (61). Integrin β1 was identified within the F5-10 WT EV proteins shared with *Cd38^-/-^* EV (**Figure 5B**). Therefore, the putative importance of Agrin/Integrin β1 interaction should be contemplated considering the total proteins identified in F5-10 WT EV (shared + unique), not only the proteins unique to F5-10 WT EV.

### 3.7 Enrichment in corona and ECM proteins in a subset of peritoneal exudate EV

**Figure 8:**
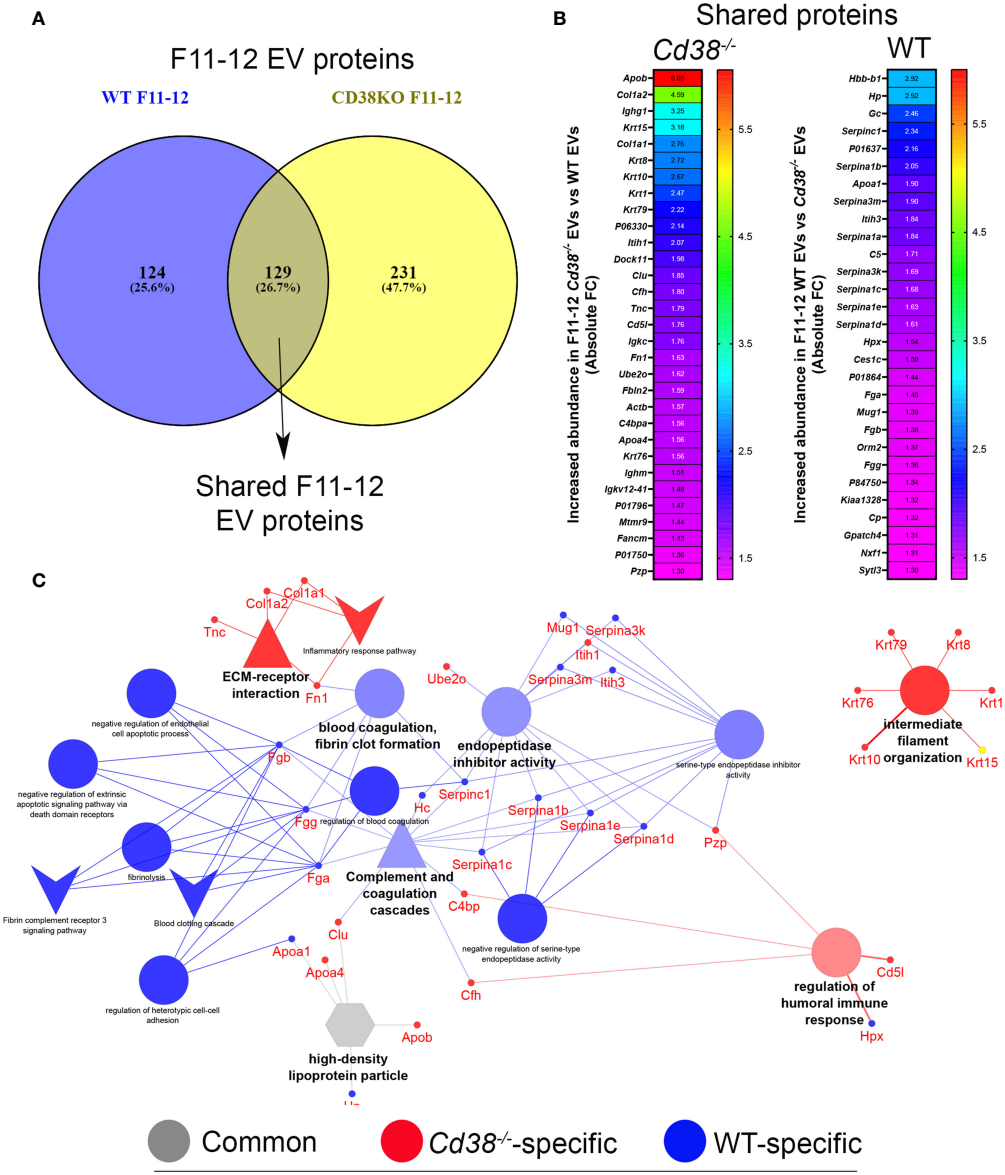
**(A)** Venn diagram highlighting the number of shared proteins identified in both peritoneal F11-12 *Cd38^-/-^* EV and F11-12 WT EV from pristane-treated mice. **(B)** Heat-maps showing the increased protein abundance in F11-12 *Cd38^-/-^* EV relative to F11-12 WT EV (left), or the increased protein abundance in F11-12 WT EV relative to F11-12 *Cd38^-/-^* EV (right). **(C)** Network of GO terms, pathways and associated genes generated from the list of shared proteins to F11-12 *Cd38^-/-^* and F11-12 WT EV, which showed an absolute FC *≥* 1.3. The network has been created using the ClueGO and Cluepedia applications of Cytoscape, with the GO Biological Process GO Cellular Component, GO Molecular Function, KEGG and Wiki Pathways; pV < 0.05. From the 2 proteins clusters that were compared, 3 categories of functional terms were generated: terms specific for F11-12 *Cd38^-/-^* EV (red), terms specific for F11-12 WT EV (blue), and common terms (grey). The node size is indicative of the term statistical significance. Terms or pathways are considered specific for a given cluster when >60% of the proteins are originating from one of the clusters. The terms with the highest statistical significance within each group are highlighted in bold and defines the name of the functional group.

The 129 common proteins to F11-12 *Cd38^-/-^* and F11-12 WT EV (**Figure 8A**) were quantitatively analyzed following a similar strategy to section 3.3. 32 proteins showed increased abundance in F11-12 *Cd38^-/-^* EV (**Figure 8B**, left), and 29 proteins had increased abundance in F11-12 WT EV (**Figure 8B**, right). As shown in **Figure 8C**, the functional analysis of common proteins with increased abundance in F11-12 *Cd38^-/-^* EV versus F11-12 WT EV showed 3 major functional groups specific for F11-12 *Cd38^-/-^* EV: ECM-receptor interaction; Regulation of humoral immune response; and Intermediate filament organization. Associated to the first 2 terms there were proteins such as collagen alpha1, collagen alpha2, Fibronectin, and proteins involved in the complement system, such as C4b-binding protein, and Complement factor H. Associated with the term intermediate filament organization there were several keratins, exclusively. In contrast, 3 functional groups were more specifically associated with F11-12 WT EV: Blood coagulation, fibrin clot formation; Endopetidase inhibitor activity; and Complement and coagulation cascades. Associated with these terms there were fibrinogens alpha, beta and gamma chains, alpha-1-antitrypsin represented by a cluster of 4 out of 6 individual Serpina1-related genes, alpha-1-antichymotrypsin represented by 2 out of 14 Serpina3-related genes, and antithrombin-III encoded by Serpinc1, the most important serine protease inhibitor in plasma that regulates the blood coagulation cascade. Some authors consider these proteins as part of the so-called protein corona, which might be characteristic for the surrounding matrix and may occur universally in biofluids (62, 63). Therefore, these results show quantitative differences in the protein cargo and functional capabilities of the ECM-enriched EV from the peritoneum of pristane-treated *Cd38^-/-^* vs WT mice. Last, the term High density lipoprotein particle was equally shared by both F11-12 pools and will be analyzed in more detail in the next section.

### 3.8 Distinct apolipoprotein profile in EV subsets

**Figure 9:**
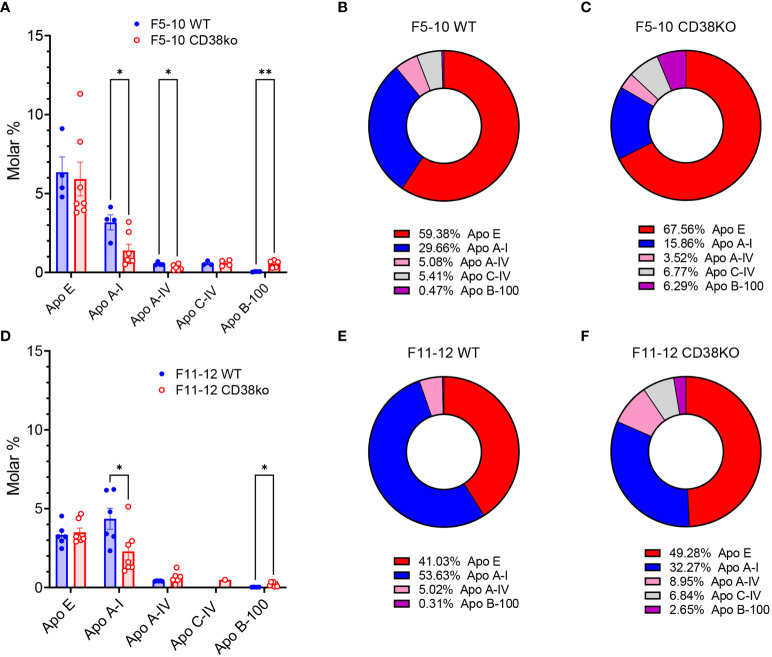
**(A)** Molar percentages of the apolipoproteins identified in F5-10 WT EV versus F5-10 *Cd38^-/-^* EV. Histograms represent the mean values, and the bars ± SEM. Unpaired t-test. Each circle represents a technical replicate from pooled biological samples. **(B)** Relative protein abundance (as percentages of total M%) of the apolipoproteins identified in F5-10 WT EV. **(C)** Relative protein abundance (as percentages of total M%) of the apolipoproteins identified in F5-10 *Cd38^-/-^* EV. **(D)** Molar percentages of the apolipoproteins identified in F11-12 WT EV versus F11-12 *Cd38^-/-^* EV. Histograms represent the mean values, and the bars ± SEM. Unpaired t-test. Each circle represents a technical replicate from pooled biological samples. **(E)** Relative protein abundance (as percentages of total M%) of the apolipoproteins identified in F11-12 WT EV. **(F)** Relative protein abundance (as percentages of total M%) of the apolipoproteins identified in F11-12 *Cd38^-/-^* EV. * = *P* < 0.05; ** = *P* < 0.01; Multiple unpaired t-test.

An interesting point in this study is the significant number of terms related with the cholesterol metabolism, most of them specific to F5-10 WT EV (**Figure 5B**), or shared by F11-12 *Cd38^-/-^* and F11-12 WT EV (**Figure 8B**). The proteins associated with these terms were the apolipoproteins Apo A-I, Apo A-IV, Apo E, and Apo B-100. Other identified protein was the phospholipid transfer protein (PLTP), which is associated with a subfraction of HDL, and may interact with the main HDL apolipoprotein, Apo A-I (64). LPPs can be distinguished from one another by the identity and amount of proteins (65). Thus, the relative abundance of the different apolipoproteins in F5-10 EV, showed that Apo E was the most abundant apolipoprotein either in M% and percentages, followed by Apo A-I, and Apo A-IV, while the mean M% for Apo B-100 was significantly low, in particular in WT EV (**Figure 9A**). In fact, Apo B-100 represented only 0.2% of total apolipoprotein content in F5-10 WT and 6.1% in F5-10 *Cd38^-/-^* EV (**Figures 9B, C**, respectively). In contrast, Apo E and Apo A-I were overrepresented as judged by their respective percentages shown in **Figures 9B, C**. Since Apo A-I is highly enriched in HDL, while Apo B-100 is specific to LDL, these results suggested a substantial enrichment in HDL relative to LDL in F5-10, in particular in F5-10 WT EV where the Apo A-I, and Apo A-IV levels were significantly higher than in F5-10 *Cd38^-/-^* EV, while for Apo B-100 occurred just the opposite. Even though, these results were unexpected given the small size of HDLs (5-10 nm) as compared with the size of F5-10 particles (**Figure 1**), and no peak at 5-10 nm was observed despite the fact the high Apo E and Apo A-I content (in M%) detected in these fractions. Similar analysis performed in F11-12 revealed a significant reduction in the abundance of Apo E, in both M% (**Figures 9D**) and percentages (**Figures 9E, F**), and a concomitant increase in the relative abundance of Apo A-I, which was more patent in F11-12 WT EV than in F11-12 *Cd38^-/-^* EV (54% vs 32% of total). In contrast, Apo B-100 represented 0.3% and 2.6% of total apoliprotein content in F11-12 WT and F11-12 *Cd38^-/-^* EV, respectively. Again, these results highlight the predominance of apolipoproteins associated to HDL particles (Apo A-I, and Apo A-IV), although the apolipoprotein profile of these 2 proteins does not correspond exactly to that of HDL particles, where Apo A-I constitutes approximately 70% of HDL protein, and is present on virtually all HDL particles (66). Moreover, Apo A-II was not detected, despite the fact that constitutes approximately 20% of HDL protein, and is present on about two-thirds of HDL particles in humans (65).

### 3.9 Validation of putative exosomal markers using a different isolation method

**Figure 10:**
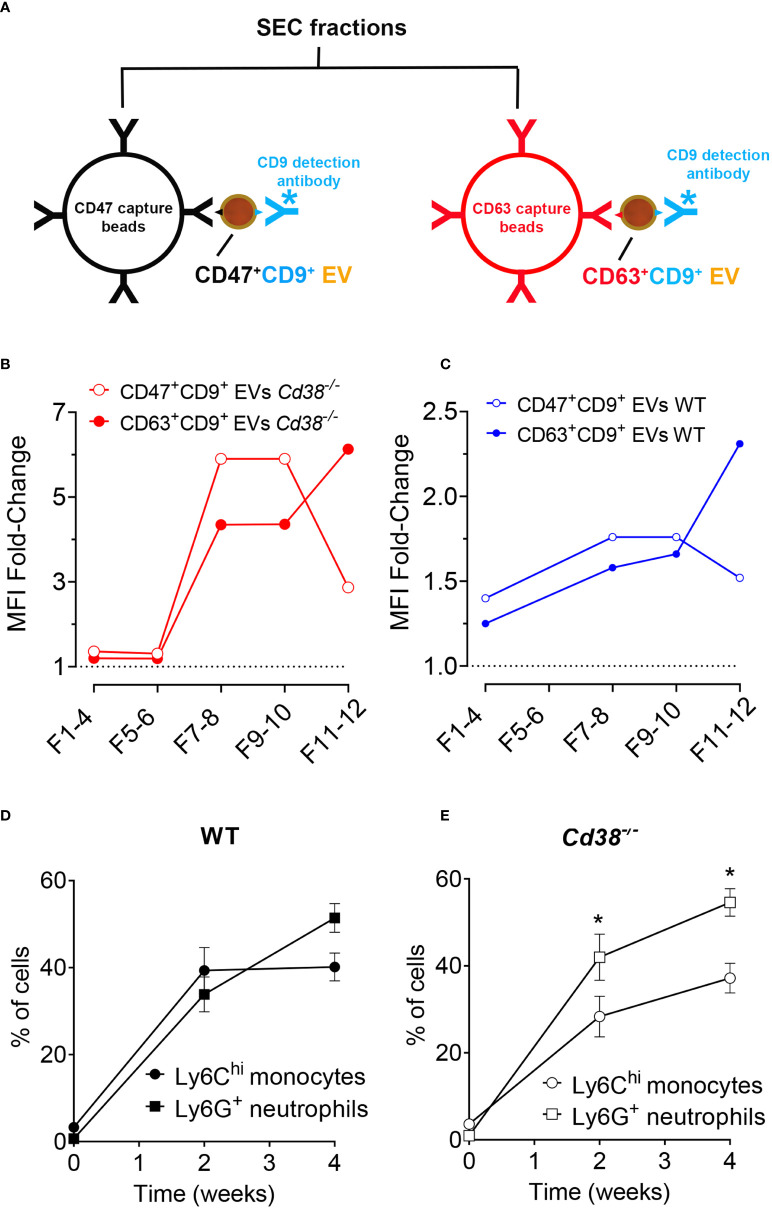
**(A)** Diagram showing the strategy used to immunocapture CD47^+^ EVs with anti-CD47-coated magnetic beads (in black), or CD63^+^ EVs (in red). Then, EV-coated beads were labeled with anti-CD9-biotin/Streptavidin-PE and analyzed by flow-cytometry. **(B)** Immunocapture of CD47^+^ EV (red solid circles) or CD63^+^ EV (open red circles) from SEC fractions of *Cd38^-/-^* EV from peritoneal exudates of pristane-treated mice. **(C)** Immunocapture of CD47^+^ EV (red solid circles) or CD63^+^ EV (open red circles) from SEC fractions of WT EV. **(D)** Time-course of frequencies of LyC^hi^ monocytes and Ly6G^+^ neutrophils in PECs from WT mice upon pristane treatment. **(E)** Time-course of frequencies of LyC^hi^ monocytes and Ly6G^+^ neutrophils in PECs from *Cd38^-/-^* mice upon pristane treatment. *Multiple unpaired t-test. Adjusted *P* value = 0.020890. Holf-Šidák method. In **(D, E)**, the number of individual samples analyzed were n = 3, before pristane treatment; n = 12, 2 weeks after pristane treatment; n = 8, 4 weeks after pristane treatment.

Proteomic analysis showed significant increased abundance of CD47 in F5-12 *Cd38^-/-^* EV versus F5-12 WT EV (**Figure 4A**), and CD47 was also detected in F5-10 *Cd38^-/-^* EV and not in F5-10 WT EV (**Figure 6B**), which indicated clear phenotypic differences between these EV. Therefore, it was of interest to validate these findings by other techniques. To this end, EV from diferent SEC fractions were immunocaptured with magnetic beads coated with either an anti-CD47 mAb, or with an anti-CD63 mAb. Then, EV-coated beads were labeled with anti-CD9-biotin coupled to Streptavidin-PE and analyzed by flow-cytometry (**Figure 10A**).

In pristane-treated *Cd38^-/-^* mice, CD47^+^ EV were detected in F7-8 and F9-10, and to a lesser extent in F11-12 (**Figure 10B**). In contrast, CD63^+^ EV were more abundant in F11-12 than in F7-8 and F9-10 (**Figure 10B**). The same occured in WT mice, although with significantly less fluourescence intensity signal than in *Cd38^-/-^* mice, which was indicative of less abundance of these EV subsets (**Figure 10C**). The immunocapture data on CD47 are consistent with the proteomic data showing significant increased abundance of CD47 in F5-10 *Cd38^-/-^* EV versus F5-10 WT EV, and relative low CD47 abundance in F11-12 versus F5-10. However, the sensitivity of the immunocapture assay seemed to be higher than proteomics, since CD63 was not detected by MS/MS in any of the pooled fractions, and CD9 was only detected in F5-10 *Cd38^-/-^* EV. The amplification of the signal provided by anti-CD9-biotin + Streptavidin-PE was remarkable. Moreover, since the capture and detecting antibodies were directed against different proteins, these assays allowed to detect EV that simultaneously expressed either CD47 and CD9 or CD63 and CD9, not those that were single positive for CD47, or single positive for CD63. Therefore, we are likely substimating the variety of EV subsets expressing either CD47, or CD63. Last, all antibodies used in these assays are directed against outer epitopes of either CD47, CD9, or CD63. Therefore, we can assume that these molecules are located at the surface of the EV in the correct orientation. In summary, the data showed that double-positive CD47^+^CD9^+^ EV were more abundant in F5-10, while CD63^+^CD9^+^ EV were more abundant in F11-12. In HeLa cells CD63 is located mainly in intracellular compartments, while CD9 is mainly at the plasma membrane but also in rare dim intracellular comparments (67). In our study, CD47 and CD9 were relatively more abundant in F5-10 than in F11-12, which correlated with increased abundance of membrane-associated proteins, and endosome-like associated proteins in F5-10 than in F11-12.

### 3.10 Comparative proteomic analyses between peritoneal exudate EV and peritoneal exudate cells revealed an EV enrichment in neutrophil proteins

**Figure 11:**
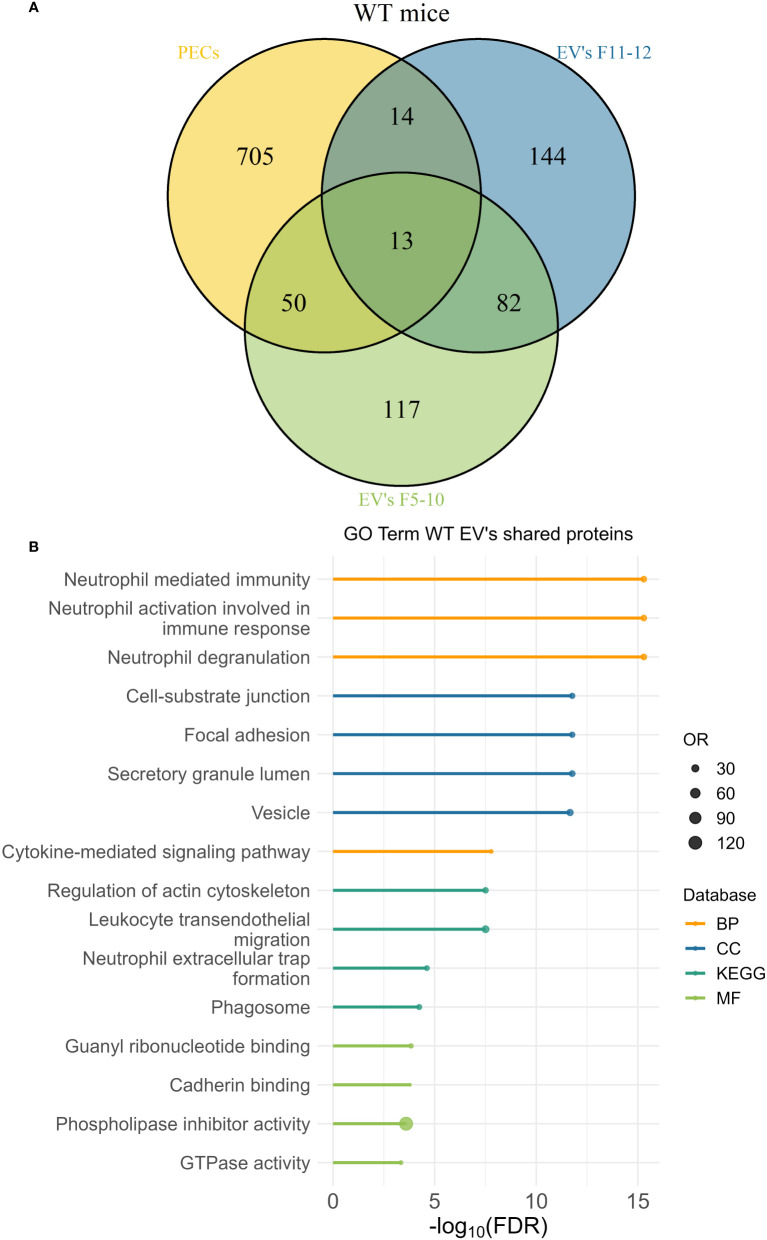
**(A)** Venn’s diagram of the proteins identified in WT PECs, F5-10 WT EV, and F11-12 WT EV. **(B)** Enrichment analysis of the proteins shared by WT PECs with F5-10 and F11-12 WT EV. The EnrichR software was used as described in Material and Methods. The –log_10_(FDR) was calculated for each database (KEGG, Biological Process, Molecular Function and Cellular Component) and the top 4 terms per each database was selected to display in the graph.

**Figure 12:**
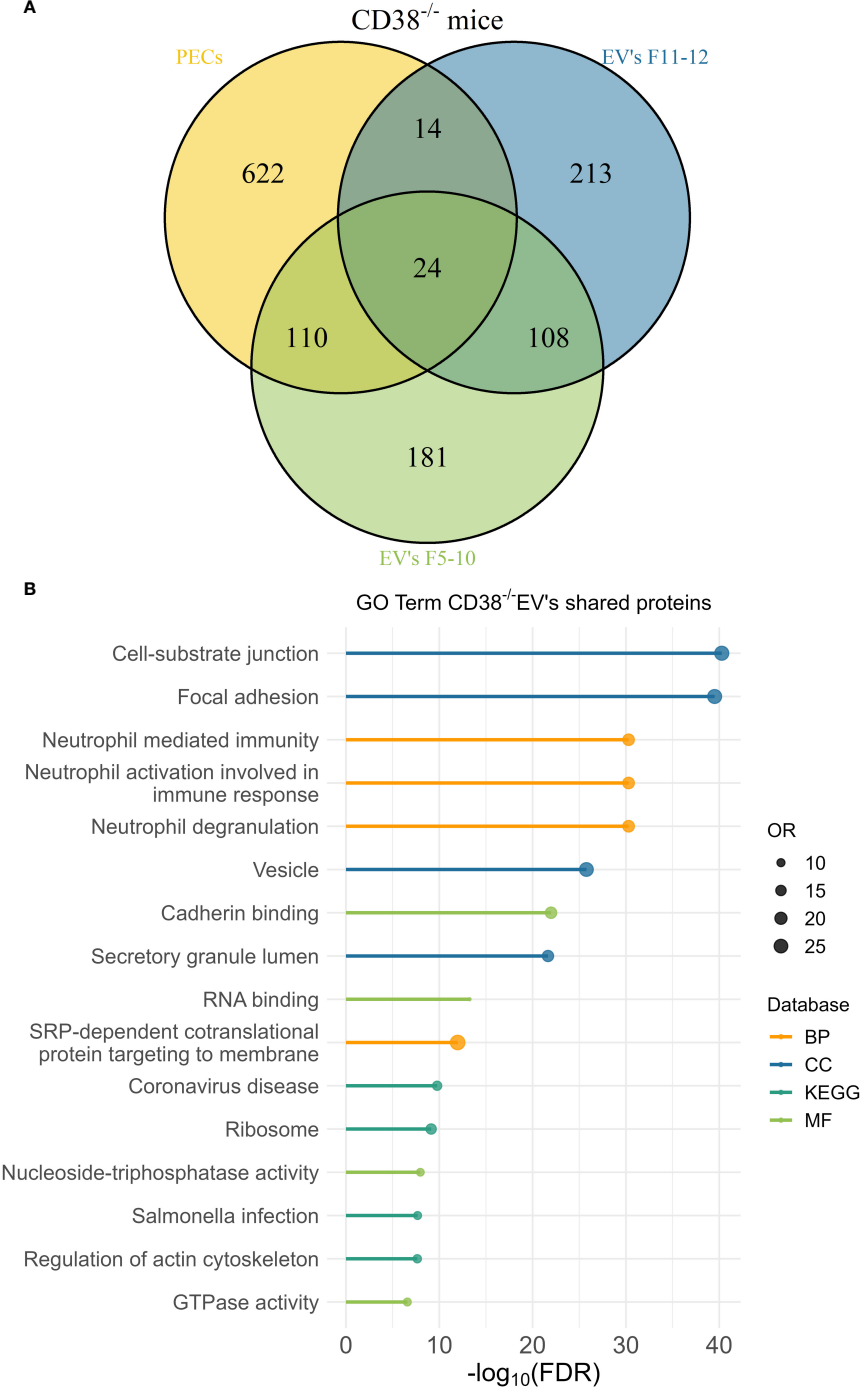
**(A)** Venn’s diagram of the proteins identified in *Cd38^-/-^* PECs, F5-10 *Cd38^-/-^* EV, and F11-12 *Cd38^-/-^* EV. **(B)** Enrichment analysis of the proteins shared by *Cd38^-/-^* PECs with F5-10 and F11-12 *Cd38^-/-^* EV. The EnrichR software was used as described in Material and Methods. The –log_10_(FDR) was calculated for each database (KEGG, Biological Process, Molecular Function and Cellular Component) and the top 4 terms per each data base was selected to display in the graph.

The peritoneal exudate of pristane-treated WT mice contained mainly inflammatory Ly6C^hi^ monocytes and Ly6G^+^ neutrophils in equal proportions (**Figure 10D**); whereas pristane-treated *Cd38^-/-^* mice contained significant higher proportion of Ly6G^+^ neutrophils than Ly6C^hi^ monocytes (**Figure 10E**), resulting in less severe inflammation (4). We analyzed whether peritoneal exudate EV and PECs shared specific protein markers that may define the cell types that were releasing those EV. First, Venn’s diagrams showed that only a fraction of the identified proteins were shared among EV and PECs, in both WT and *Cd38^-/-^* EV (**Figures 11A**, **12A**, respectively). EnrichR analyses of the shared proteins were performed. Interestingly, the top 3 BP functional terms were related with neutrophil functions: *neutrophil degranulation* (GO:0043312), *neutrophil activation involved in immune response* (GO:0002283), and *neutrophil mediated immunity* (GO:0002446), either in WT EV (**Figure 11B**), or in *Cd38^-/-^* EV (**Figure 12B**). These GO terms comprised 22 proteins in WT EV (adjusted *P* value = 5.07E-16) versus 44 proteins in *Cd38^-/-^* EV (adjusted *P* value = 5.19E-31), which was indicative of stronger neutrophil protein signature in *Cd38^-/-^* EV.

**Figure 13:**
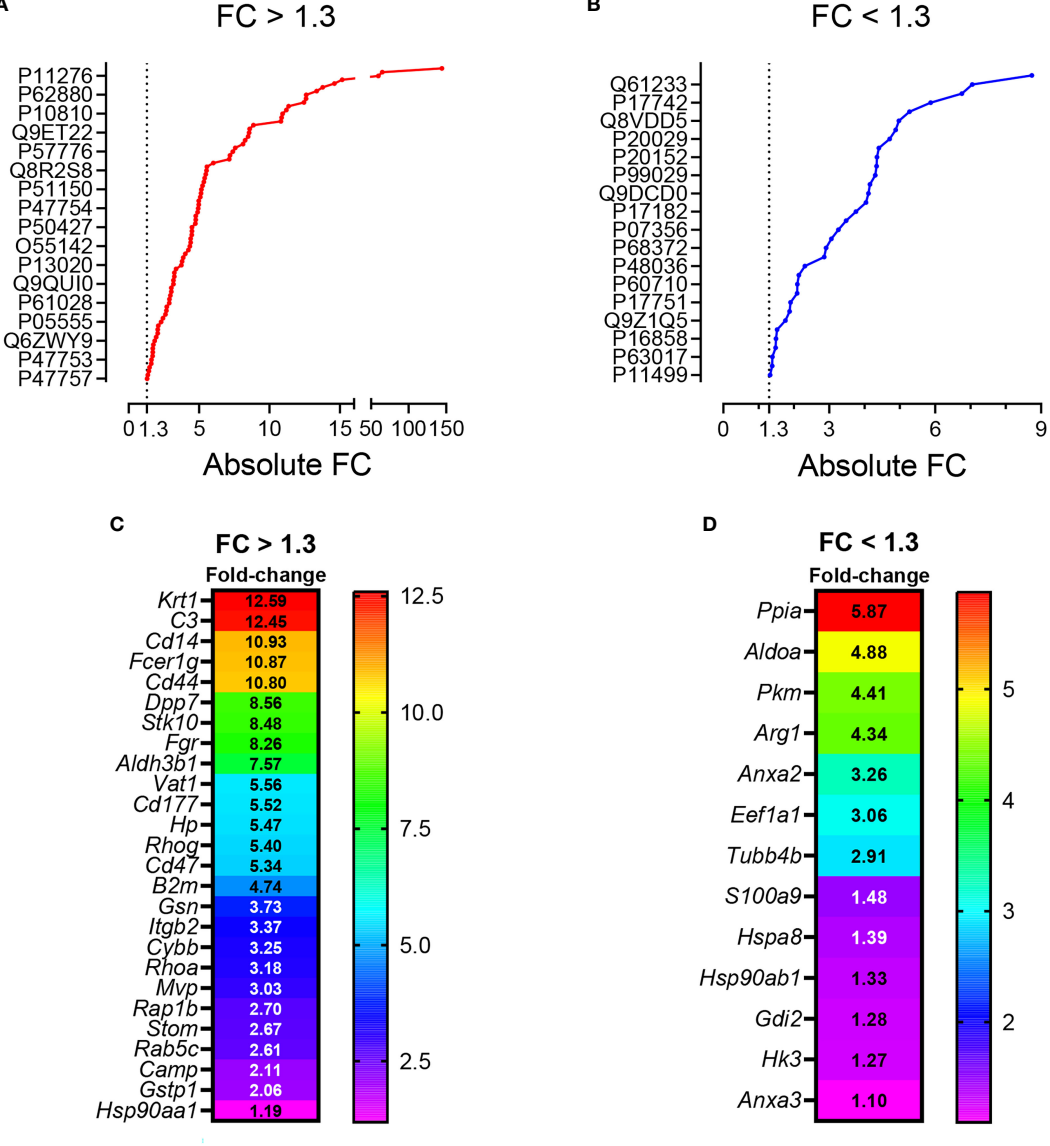
**(A)** Plot showing the proteins with increased abundance in F5-10 *Cd38^-/-^* EV relative to *Cd38^-/-^* PECs. **(B)** Plot showing the proteins with decreased abundance in F5-10 *Cd38^-/-^* EV relative to *Cd38^-/-^* PECs. In **(A, B)** the absolute Fold-Change (FC) in abundance (X-axis) was calculated as described in Material and Methods and Results sections. A cutoff of 1.3 fold was selected for biological significance (dotted lines). In both panels, each dot represents one single protein. However, the protein IDs shown in the Y-axis of **(A, B)** are representing just only 17 out of the total number of proteins in each group. The selection was done automatically by the GraphPad Prism software based on the available space. **(C)** Heat map representing the absolute FC values of the neutrophil signature EV proteins with increased abundance relative to *Cd38^-/-^* PECs. **(D)** Heat map representing the absolute FC values of the neutrophil signature EV proteins with decreased abundance relative to *Cd38^-/-^* PECs. In **(C, D)** names of the codifying genes are shown (left rows). Neutrophil signature EV proteins with an absolute FC below 1.3 are also shown.

We then focused our interest on the protein abundance profiles of F5-10 *Cd38^-/-^* EV vs *Cd38^-/-^* PECs, since they share the largest number of proteins of the analyzed samples, and therefore, their numbers are reliable to perform further statistical analysis. The comparison of the molar percentages of the shared proteins in PECs versus F5-10 EV clearly indicated that many of the F5-10 EV proteins showed increased abundance relative to their counterparts in PECs, although there were also a significant number of them with decreased abundance relative to that in PECs (data not shown). To better appreciate these differences, the fold-change (FC) in protein abundance was calculated by dividing the molar percentage (M%) value of an individual protein in F5-10 *Cd38^-/-^* EV with the cognate value in *Cd38^-/-^* PECs. The absolute FC value was then calculated as in section 3.4. The cutoff value of 1.3 was then used to separate the proteins in two categories or subsets: 83 out of 134 shared proteins showed increased abundance in F5-10 *Cd38^-/-^* EV relative to that in *Cd38^-/-^* PECs (**Figure 13A**), while 34 out of 134 showed decreased abundance (**Figure 13B**). To assess whether the EV proteins of the neutrophil signature revealed by the EnrichR analysis showed increased or decreased abundance relative to their expression in PECs, the proteins lists shown in **Figure 13A** and **Figure 13B** were matched for coincidences with the proteins of the neutrophil signature list. The majority of proteins of the neutrophil signature (25 out of 39; 64.10%) were among the F5-10 *Cd38^-/-^* EV proteins with increased abundance relative to PECs (**Figure 13C**), while only 10 out 39 (25.64%) were among the proteins with decreased abundance in F5-10 *Cd38^-/-^* EV (**Figure 13D**). Another 4 proteins were in the absolute FC range below 1.3. Therefore, the protein profile of the neutrophil signature reflected the protein abundance profile of the 134 proteins shared with PECs. Overall, these results provide quantitative evidence of a significant enrichment in neutrophil-derived proteins in peritoneal exudate *Cd38^-/-^* EV from pristane-treated mice.

To further assess the differences in protein composition between peritoneal exudate EV and their corresponding PECs we performed Cellular Component enrichment analyses in F5-10 EV, F11-12 EV and PECs using the ClueGO app. On average only 1-2% of the identified EV proteins were of mitochondrial origin, either in *Cd38^-/-^* or WT EV, which resulted in the virtual absence of mitochondrial-related GO terms (data not shown). In contrast, in PECs 23-24% of the identified proteins were of mitochondrial origin, and the GO term *mitochondrion* was the top second CC group term. This term showed highly statistical significance in both *Cd38^-/-^* PECs (170 proteins were categorized within the term mitochondrion, which resulted in an adjusted pValue for this term = 5.09E-37), and WT PECs (n = 182, adjusted pValue = 1.13E-33). These data revealed strong depletion of mitochondrial proteins in EV relative to PECs. According with other authors, mitochondrial proteins are absent from small EV isolated by ultracentrifugation (68). Overall, these observations indicate that the peritoneal exudate EV have a protein composition qualitative and quantitatively different from that in whole PECs.

## 4 Discussion

In this study we report, for the first time to our best knowledge, the presence, isolation and characterization of EV from peritoneal exudates of pristane-treated mice. SEC-isolated F5-10 and F11-12 EV had a size and morphology compatible with small EV, as shown by NTA and TEM analyses, and following the MISEV2018 guidelines (8). Both F5-10 and F11-12 EV contained tetraspanin proteins, with increased abundance of CD9 in F5-10 EV, and CD63 in F11-12 EV. Another tetraspanin, CD82 was only detected in F5-10 WT EV. In contrast, F5-10 EV contained a significant number of transmembrane (TM) proteins, including 1-TM proteins such as integrins, or class II molecules, 5-TM proteins as CD47, or 7-TM proteins as CD88, which were not present in F11-12 EV. Moreover, F5-10 EV showed a larger number of proteins associated to membrane than in F11-12 EV, which include a number of trimeric G proteins, and small G proteins. Thus, small G proteins such as Rab5c and Rab7a, which are involved in early endosome to late endosome transport, are particularly enriched in F5-10 EV vs F11-12 EV from CD38-deficient mice. In contrast, Rab27a, which controls exosome secretion in human cells (69, 70), was selectively enriched in F11-12 *Cd38^-/-^* EV (by Western-blot analysis, data not shown). Likewise, GO cellular component terms such as melanosome, extracellular exosome, early endosome, plasma membrane raft, lamellipodium, and cytoplasmic side of membrane were enriched in F5-10 EV vs F11-12 EV.

According with our immunocapture data, the CD63^+^CD9^+^ EV, which are predominant in F11-12 fractions, and therefore, are smaller than F5-10 EV, may represent exosomes enriched in late endosome components that are defined as a small EV subtype bearing the tetraspanin CD63 in HeLa cells (67), while in F5-10 EV predominate CD47^+^CD9^+^ EV, which are likely representative of a distinct EV subset with different functional capabilities (71–74). CD47-enriched EV inhibit dendritic cell activation and ameliorate hepatic ischemia-reperfusion injury *via* interaction with SIRPα/CD172*α* ^+^ DCs (75). CD47-enriched exosomes have been used to evade phagocytosis by the mononuclear phagocytic system for anticancer therapy (76). CD47 functions as a “don’t eat me signal” by binding to inhibitory SIRPα and thereby inhibits certain effector functions of neutrophils and macrophages, including host cell phagocytosis, transendothelial migration, and phagocyte NADPH oxidase activity (77). Whether CD47^+^ EV exert similar functions in pristane-treated mice requires further investigation. We have also identified increased abundance of another CD47 ligand, thrombospondin-1 (TSP-1 encoded by *Thbs1*), in F5-12 *Cd38^-/-^* vs F5-12 WT EV. TSP-1 was reported to promote the resolution of inflammatory process and to facilitate phagocytosis of damaged cells (37), and TSP-1 restricts IL-36γ-mediated neutrophilic inflammation during *Pseudomonas aeruginosa* pulmonary infection (78). Therefore, TSP-1 may play anti-inflammatory and immunoregulatory roles in SLE autoimmunity.

The above data are indicative of the heterogeneity of the small EV isolated from peritoneal exudates from pristane-treated mice. On one hand, there are proteins compatible with the presence of endosome-derived EV (exosomes), whereas the quantitative proteomics data strongly suggest the predominance of small EV of ectosomal origin in F5-10 EV, as defined after the proteomic studies performed in monocyte-derived DCs (68), and biogenesis studies performed in human HeLa cells (67). In this sense, elegant studies performed by Jeppesen et al., identify the membrane-associated proteins Annexin A1 and A2 as novel markers of non-exosomal small to large EV and 150–1,000 nm Annexin A1-positive microvesicles budding off the plasma membrane (57). In our study, Annexin A1 and Annexin A2 proteins were identified in both F5-10 *Cd38^-/-^* EV and WT EV. While Annexin A1 shows increased abundance in F5-10 *Cd38^-/-^* EV vs F5-10 WT EV, Annexin A2 shows equal abundance. In human PMNs, Annexin A1 represents between 2% and 4% of total cytosolic proteins, it is considered as an anti-inflammatory protein (79), and microparticles bearing Annexin A1 can indeed elicit rapid nongenomic anti-inflammatory and homeostatic effects (79). Elevated expression of this protein in neutrophil EV may be indicative of a nonpathogenic neutrophil activation, as observed in microparticles obtained from skin blister exudates, a self-resolving inflammatory model characterized by a highly neutrophilic inflammation (80).

The pro-apoptotic effect of CD38 and TRPM2 expression in Ly6C^hi^ monocytes is particularly harmful in the pristane-induced model of lupus in which PECs lack highly phagocytic resident peritoneal macrophages (Tim4^+^ macs), and have low numbers of anti-inflammatory elicited macrophages (Tim4^−^CD138^+^Marco^+^ macs), which have increased phagocytic capacity for apoptotic cell clearance compared to Tim4^+^ macs (81). Macrophages are the primary cell type responsible for the resolution of inflammation and the clearance of apoptotic debris in most tissues. Phagocytosis of apoptotic neutrophils by macrophages promotes a switch from a pro- to an anti-inflammatory macrophage phenotype (82), which is clearly delayed or absent in pristane-induced lupus and other animal models of lupus (83). Although it has not been experimentally proven, it has been proposed than in pristane-treated mice the apoptosis of peritoneal neutrophils may contribute to the resolution of the chronic inflammation by inducing the development of anti-inflammatory macrophages and generating Annexin A1 and other mediators (7). We have not observed increased neutrophil apoptosis at 2 weeks after pristane injection (4), which makes unlikely that the source of Annexin A1^+^ EV are derived from dying neutrophils. It is more likely that the release of Annexin A1^+^ EV by neutrophils or other cells may occur prior to the proposed pristane-induced apoptosis of neutrophils (7). In fact, the release of Annexin A1 may potentiate the apoptosis of neutrophils, facilitating the resolution of inflammation (84). F5-10 *Cd38^-/-^* EV are also enriched in Arginase-1, and therefore, may exert inhibitory functions on T cells, as it occurs with small EV found in the ascites and plasma of ovarian cancer patients (85). However, the abundance of Arginase-1 in F5-10 *Cd38^-/-^* EV is similar to that in F5-10 WT EV, and therefore, it is not expected in these EV a distinct Arginase-1-dependent functional effect. However, Arginase-1, together with Annexin A1, CD47, TSP-1, and Sirpα1, may represent a potent set of proteins with anti-inflammatory characteristics that are enriched in F5-10 *Cd38^-/-^* EV. Overall, these results reveal a mechanism by which neutrophils may contribute to the resolution of pristane-induced chronic inflammation by releasing Annexin A1^+^ EV, which may regulate macrophage cell responses in otherwise a strong pro-inflammatory environment.

F5-10 *Cd38^-/-^* EV and F5-10 WT EV share a number of terms/pathways, which are represented by proteins highly expressed in neutrophils. Thus, CD177 is specific for a subset of neutrophils, which is increased significantly in SLE patients (86). It is a glycosyl-phosphatidylinositol (GPI)-anchored glycoprotein, first described in 1971 as the NB1 antigen and a member of the leucocyte antigen 6 superfamily (87). Functionally interacts with CD11b/CD18 heterodimers (88). On the other hand, Fcer1g is functionally linked to CD18-mediated neutrophil activation (89). In this sense, the integrins CD18 and CD11b are associated with a larger number of terms and functional groups, including many of the terms specific for CD177, or Fcer1g. It is interesting to note that CD14, which is considered a monocyte marker, it is also expressed in neutrophils and its surface expression can be up-regulated from intracellular stores with immunomodulators such as formyl peptides and IFN-γ (90). To note, CD14 is present in both plasma-membrane secretory vesicles and azurophilic granules (91), while CD177 is located in specific granules and CD11b/CD18 (Mac-1) in gelatinase granules (92), while many other of the identified proteins are located in secretory granules, either in the lumen (Hspa8, C3b), or at the plasma membrane (H2-D1, TLR2, CD44, Rab proteins). These results were indicative of EV enriched in proteins derived from neutrophils. In support of the selective enrichment in neutrophil-derived proteins was the massive recruitment of neutrophils to the PC that occurs in mice after two weeks of i.p. injection of pristane (4). The proteomics quantitative data indicate that these proteins are more abundant in F5-10 *Cd38^-/-^* EV than in WT EV, and this correlated with the higher proportion of neutrophils than Ly6C^hi^ monocytes found in the PC of pristane-treated *Cd38^-/-^* mice (**Figure 10**). Neutrophil EV are heterogeneous in protein composition and in protein abundance, and can deliver packaged information propagating the activation status of the parent cells (80).

In F11-12 EV we have observed an enrichment in a number of proteins which are in dispute whether or not they are truly components of EV or just contaminants from the biological fluids where these EV were isolated. Toth et al., identified nine shared EV corona proteins (Apo A-I, Apo B, Apo C-III, Apo E, complement factors 3 and 4B, fibrinogen α-chain, immunoglobulin heavy constant γ2 and γ4 chains), which appear to be common corona proteins among EV, viruses and artificial nanoparticles in blood plasma (63). By using extensive and quantitative proteomic analyses, other authors have shown in human monocyte-derived dendritic cells the presence of a subtype of high-density small EV enriched in extracellular matrix (fibronectin, collagen) and serum derived proteins (albumin, complement), but not in late endosome-associated proteins (68). In contrast, the lighter fraction of the same pellet was the only fraction presenting enrichment in both plasma membrane and late-endosomal proteins, thus the only fraction containing *bona fide* exosomes (in addition to other small EV) (68). Therefore, according with Kowal’s classification of small EV (68), it is likely that F11-12 contains light-density CD63^+^ exosomes, which are a minority as judged by their protein abundance, and high-density small EV enriched in ECM and coagulation/complement proteins, with important functional capabilities (93). The ECM proteins, complement proteins, serum albumin, and several apolipoproteins may be also components of the protein corona that presumably should be associated with any type of EV.

The origin of the ECM proteins detected in F11-12 EV is unknown. Some data are indicative that the ECMs may be released from mesothelial cells or other cells present in the submesothelium, which are subjected to an extraordinary stress because the ongoing inflammatory process. The membrane that lines the abdominal cavity and all peritoneal organs is formed by a monolayer of peritoneal mesothelial cells (PMCs) with epithelial characteristics, that rests on a thin basal membrane and an underlying stroma composed of ECM and connective tissue with few capillaries and resident fibroblasts (94). However, mesothelial cells have the ability to change their phenotype comparable to changes seen in epithelial-to-mesenchymal transition (EMT), which has been termed mesothelial-to-mesenchymal transition (MMT) (95). This has implications both in normal repair and pathological processes (94, 96, 97). PMCs participate in initiating and resolving serosal inflammation and repair by secreting various pro-, anti- and immunomodulatory mediators into the serosal fluid and submesothelial compartment. These include products of the coagulation cascade, chemokines, cytokines and growth factors, prostaglandins and prostacyclin, reactive nitrogen and oxygen species, antioxidant enzymes and ECM molecules including collagen types I, III, and IV, elastin, fibronectin, laminin, and proteoglycans (98, 99). Many of these proteins are indeed detected in F11-12 EV. All these proteins are related with known mesothelial cells functions such as remodeling and ECM expansion, and fibrin deposition, which are altered under inflammatory conditions (99). The fibroblast-like characteristics induced in PMCs that have undergone MMT allow them to invade into the submesothelial stroma or into the intraperitoneal ascitic fluid, where the cells or their secreted EV may contribute to perpetuate or resolve the inflammatory process depending upon their protein cargo. It is therefore likely that many peritoneal exudate proteins derived from PMCs or fibroblast-like cells are on the surface of small EV released by these or other cells, with a variable protein cargo depending upon the inflammatory conditions. Thus, cytokeratins, which are used as epithelial markers of PMCs (96), fibronectin and Type I collagens are more abundant in F11-12 *Cd38^-/-^* EV than in F11-12 WT EV, whereas Fibrinogen alpha, beta, and gamma chains are more abundant in the latter. It is also likely that the protein corona may provide to these EV functional capabilities that lack EV devoid of the protein corona (63).

Regarding the presence of other nanoparticles, such as LPPs, it is important to note that although Apo E content in human HDL is low, in mice and in other species that constitutively lack cholesteryl ester (CE) transfer protein (CETP) (100), HDL presents a prominent subclass that is enriched in apoE, referred to as HDL_1_, HDL_c_, or HDL-with Apo E (101). These Apo E-enriched HDLs have the capacity to accept free cholesterol from macrophages, which is then esterified by the enzyme lecithin:cholesterol acyltransferase (LCAT) resulting in HDL particles of increased size and lower density (102). In our study, Apo E enrichment relative to Apo AI is 4-fold in F5-10 *Cd38^-/-^* EV and about 2-fold in F5-10 WT EV, which suggests that a large amount of Apo E detected in these fractions is not associated with HDL particles. In fact, the GO cellular component *early endosome* found enriched in F5-10 *Cd38^-/-^* EV included Apo E among the 20 associated proteins, and not other apolipoproteins. In this sense, there is evidence of the association of Apo E with ILVs and exosomes of pigment cells, which is required for the ESCRT-independent sorting mechanism of PMEL onto ILVs and the endosomal formation of PMEL amyloid fibrils (103). The presence of Apo E in exosomes secreted by other cell types such as oligodendrocytes, and hepatocytes (104, 105) suggests a widespread mechanism where Apo E could act as a general loading mechanism for soluble proteins or shed luminal domains of endosomal transmembrane proteins (103). Therefore, our results are compatible with an increased abundance of Apo E in either EV (F5-10) and HDL particles (F11-12). Whether these HDL particles, or their protein components may directly interact with the EV forming part of the protein corona (63) remains to be investigated.

## 5 Conclusions

**Figure 14:**
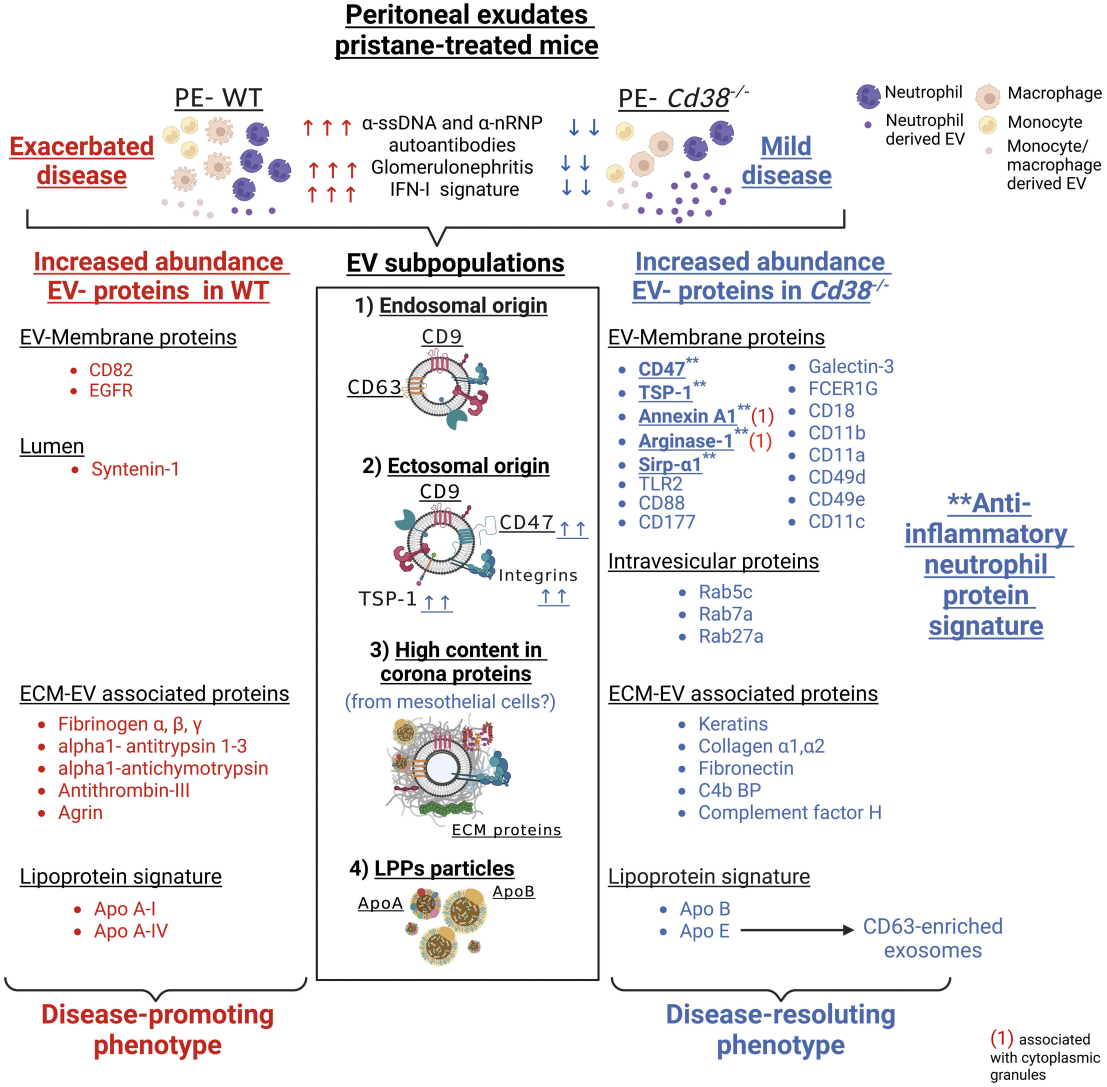
Schematic representation of the major findings of this study. Proteins were assigned to the different types of EV and lipoprotein particles present in peritoneal exudates from pristane-treated WT mice (highlighted in red, left) versus CD38-deficient mice (highlighted in blue, right), as suggested by the quantitative proteomic comparison analyses. Syntenin-1, which is considered by some authors as a putative universal exosome marker, was exclusively detected in F5-12 WT EVs (crude data available in ProteomeXchange repository, see below). Only a fraction of the proteins, which are part of the neutrophil protein signature, are shown. ** highlights the proteins of the anti-inflammatory neutrophil protein signature, which have been considered of special importance in the discussion section. (1) These cytosolic proteins are associated with cytoplasmic neutrophil granules.

The field of proteomics includes very powerful techniques that allow to know on a large scale, together with transcriptomics and metabolomics, the pathological circumstances of a living organism. In this study, thanks to an extensive proteomic analysis and powerful bioinformatics software, distinct EV subtypes were identified in the peritoneal exudates of pristane-treated mice (**Figure 14**): 1) small EV enriched in the tetraspanins CD63 and CD9, which are likely of exosomal origin; 2) small EV enriched in CD47 and CD9, which are also enriched in plasma-membrane and membrane-associated proteins, with an ectosomal origin; 3) small EV enriched in keratins, ECM proteins, complement/coagulation proteins, fibrin clot formation proteins, and endopetidase inhibitor proteins. This enrichment may have an inflammation-mediated mesothelial-to-mesenchymal transition origin, representing a protein corona on the surface of peritoneal exudate EV; 4) HDL-enriched lipoprotein particles. Quantitative proteomic analysis allowed us to identify an anti-inflammatory neutrophil protein signature, which was more prominent in EV from pristane-treated *Cd38^-/-^* mice, and quantitative differences in the protein cargo of the ECM-enriched EV from *Cd38^-/-^* vs WT mice. These differences are likely to be related with the distinct inflammatory outcome shown by *Cd38^-/-^* vs WT mice in response to pristane treatment, although further analyses are required to reinforce this interpretation. Our results demonstrate the power of a hypothesis-free and data-driven approach to transform the heterogeneity of the peritoneal exudate EV from pristane-treated mice in valuable information about the relative proportion of different EV in a given sample and to identify potential protein markers specific for the different small EV subtypes, in particular those proteins defining EV involved in the resolution phase of chronic inflammation.

## Data availability statement

The mass spectrometry proteomic data presented in the study are deposited to the ProteomeXchange Consortium via the PRIDE partner repository (https://www.ebi.ac.uk/pride/archive/) with the dataset identifiers PXD03527 and 10.6019/PXD03527.

## Ethics statement

The animal study was reviewed and approved by CSIC Ethics Committee. Consejo Superior de Investigaciones Científicas. Madrid. Spain.

## Author contributions

PC-R, J-AR-G, and AC-P have performed experimental work, and data analyses. They have contributed equally to this work and share first authorship. MP-P, EG-P, CF-H, PR-C-T, AM-C, and M-MC-C have performed experimental work and data analyses. LM-H performed microscopy imaging analyses. AL and VL performed the proteomics analyses. MP-S-C and SR-S performed the flow cytometry analyses. A-BJ performed the NTA analyses of EV. FB participated in the NTA analysis and characterization of the EV. EZ performed the initial experiments and contributed to the interpretation of the results. RM performed the autoantibody and total IgG analyses and contributed to the interpretation of the results. JS and MZ, corresponding authors, contributed to the conception and design of the study, organized the database, and performed some of the experimental work. JS organized the first draft of the manuscript. MZ wrote sections of the manuscript. All authors contributed to the article and approved the submitted version.

## Funding

Financial support: JS and MZ: Grant SAF-2017-89801-R (Proyecto del plan estatal, Ministerio de Ciencia e Innovación). RM: Grant: PID2020-119567RB-I00. The Proteomic Unit of the IPBLN-CSIC belongs to Proteo-Red-ISCIII (PRB2 and PRB3) and is supported by grants PT13/0001/011 and PT17/0019/0010 CSIC supported in part the article processing charges.

## Acknowledgments

We thank to Dr. Frances E. Lund (Department of Microbiology, University of Alabama at Birmingham (UAB), AL, USA), for the gift of the *Cd38^-/-^* mice. We thank to Clara Sánchez-González, Jorge Huertas-Latorre and Francisco Ferrer-Gamarra for their contribution with the mice experimental procedures at the IPBLN-CSIC Animal Facility, Granada, Spain. We thank to Yesica Molina-Castro, Biotecnology Grade student at UGR, and Sandra Garcia-Jimenez, student at Campus Formation for their contribution to the success of specific experiments. We thank to Dr. David Porcel Muñoz, Department of TEM, Centro de Instrumentación Científica (CIC), UGR for his contribution in the TEM experiments. **Figure 14** was created in BioRender.com.

## Conflict of interest

The authors declare that the research was conducted in the absence of any commercial or financial relationships that could be construed as a potential conflict of interest.

## Publisher’s note

All claims expressed in this article are solely those of the authors and do not necessarily represent those of their affiliated organizations, or those of the publisher, the editors and the reviewers. Any product that may be evaluated in this article, or claim that may be made by its manufacturer, is not guaranteed or endorsed by the publisher.
