# Supplementary material for: Extracellular vesicles from pristane-treated CD38-deficient mice express an anti-inflammatory neutrophil protein signature, which reflects the mild lupus severity elicited in these mice
Source: Front Immunol. 2022 Oct 24;13:1013236. doi: 10.3389/fimmu.2022.1013236 (PMC9638146; doi:10.3389/fimmu.2022.1013236)
Supplement: Supplementary file 1 [file DataSheet_1.pdf]

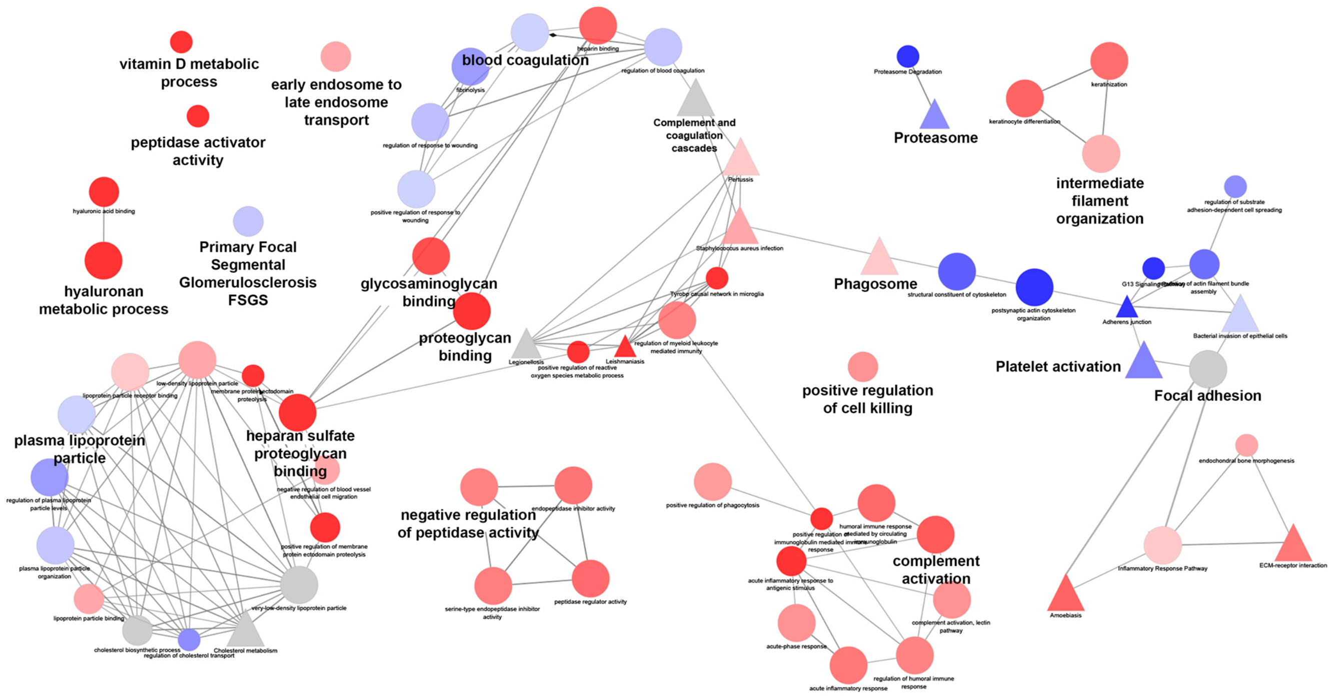

Figure S1. Functional network created from 140 proteins common to F5-12 Cd38⁻/⁻ EVs and F5-12 WT EVs that showed an absolute FC ≥ 1.3. The network has been created using the ClueGO and Cluepedia applications of Cytoscape, with the GO Biological Process (elipses), GO Cellular Component (hexagons), GO Molecular Function (rectangles), KEGG (triangles), and Wiki Pathways (V shape); pV < 0.05. From the 2 proteins clusters that were compared, 3 categories of functional terms were generated: terms specific for F5-12 Cd38⁻/⁻ EVs (red), terms specific for F5-12 WT EVs (blue), and common terms (grey). The node size is indicative of the term statistical significance. Terms are considered specific for a given cluster when >60% of the proteins are originating from one of the clusters. The term with the highest statistical significance within each functional group is highlighted in bold and defines the name of the group.
